# Highly Stretchable, Self‐Healable, and Conductive Gelatin Methacryloyl Hydrogel for Long‐Lasting Wearable Tactile Sensors

**DOI:** 10.1002/advs.202502678

**Published:** 2025-05-29

**Authors:** Zhikang Li, Bin Wang, Jijian Lu, Yumeng Xue, Jiaxiang Wang, Boqing Jia, Gengyu Han, Yihe Zhao, Muhammad Afzal Khan Qureshi, Lan Yu, Kang Zhao, Min Li, Ping Yang, Dejiang Lu, Libo Zhao

**Affiliations:** ^1^ State Key Laboratory for Manufacturing System Engineering International Joint Laboratory for Micro/Nano Manufacturing and Measurement Technologies State Industry‐Education Integration Center for Medical Innovations Xi'an Jiaotong University (Yantai) Research Institute for Intelligent Sensing Technology and System Xi'an Jiaotong University Xi'an 710049 China; ^2^ Shandong Laboratory of Yantai Advanced Materials and Green Manufacturing Yantai 264000 China; ^3^ School of Mechanical Engineering Xi'an Jiaotong University Xi'an 710049 China; ^4^ School of Instrument Science and Technology Xi'an Jiaotong University Xi'an 710049 China; ^5^ State Key Laboratory of Solidification Processing School of Materials Science and Engineering Northwestern Polytechnical University and Shaanxi Joint Laboratory of Graphene Xi'an 710072 China; ^6^ Department of Orthopaedics Peking University Third Hospital Beijing 100191 China; ^7^ Engineering Research Center of Bone and Joint Precision Medicine Peking University Third Hospital Beijing 100191 China; ^8^ Beijing Key Laboratory of Spinal Disease Research Peking University Third Hospital Beijing 100191 China

**Keywords:** gelatin methacryloyl (GelMA) hydrogel, ionic conductivity, self‐healing, stretchability, wearable strain sensors

## Abstract

Constructing hydrogels with both remarkable mechanical and self‐healing properties is highly desirable for soft electronics, yet remains challenging due to conflicting demands on chemical bonds and polymer chain mobility. Herein, a highly stretchable, self‐healing, and conductive gelatin methacryloyl (GelMA) hydrogel is developed by incorporating polyvinyl alcohol, N‐(2‐amino‐2‐oxoethyl)‐2‐propenamide, sodium tetraborate, and sodium chloride into GelMA, followed by a two‐step polymerization process. The introduced novel interpenetrating networks, hierarchical hydrogen bonds (weak and strong H‐bonds), and borate ester bonds (BEBs) synergistically improve the mechanical strength, and concurrently function as sacrificial bonds for energy dissipation under deformation. Moreover, the constructed reversible BEBs and weak H‐bonds enable autonomous self‐healing at room temperature. The resulting hydrogel achieves remarkable stretchability (≈160%), tensile strength (≈130 kPa), and self‐healing efficiency (86%), surpassing previously reported GelMA hydrogels. Importantly, a self‐healing GelMA hydrogel strain sensor is demonstrated, featuring a high gauge factor (≈3.28), ultra‐low detection limit (0.1%), and excellent recovery of sensitivity (≈100%) and detection range (≈75%) after damage. Successful monitoring of subtle and large‐scale human motions with both original and healed sensors highlights the device's durability and longevity. This study provides a promising approach for the rational design and practical application of GelMA hydrogels in wearable bioelectronics.

## Graphical abstract

Gelatin methacryloyl hydrogel, which possesses high stretchability, tensile strength, toughness, and autonomous self‐healing capability at room temperature, is developed through the introduction of interpenetrating networks, hierarchical hydrogen bonds (weak and strong H‐bonds), and borate ester bonds. It is synthesized via a two‐step polymerization process, and utilized for wearable self‐healing strain sensors, demonstrating excellent performance recovery ability after damage in various human motions monitoring.

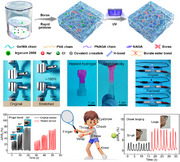

## 1 Introduction

Hydrogel‐based soft electronics have demonstrated immense potential in skin‐wearable and implantable medical applications, and garnered extensive research in recent years.^[^ ^1^, ^2^, ^3^ ^]^ The tissue‐mimicking Young's modulus (from a few pascals to several megapascals), exceptional biocompatibility, and biologically similar ionic conductivity of hydrogels render the soft electronics with excellent bio‐mechanical matching at device‐tissue interfaces, biological safety (minimal immune response), and readily electrical communication with biological tissues. Given these merits, a diversity of hydrogel‐based electronics, such as wearable sensors,^[^ ^4^, ^5^, ^6^ ^]^ soft actuators,^[^ ^7^, ^8^, ^9^ ^]^ wound dressings,^[^ ^10^, ^11^ ^]^ and implantable tissue/organ patches,^[^ ^12^, ^13^ ^]^ have been developed and successfully applied to physiological signals monitoring, wound treatment, and neuromodulation, unveiling tremendous potential.^[^ ^14^, ^15^ ^]^ Despite these successes, the majority of hydrogel soft electronics still face crucial challenges: 1) they are susceptible to mechanical damage such as scratch, wear, tear, and puncture, during their use process because of their low mechanical strength, severely hindering their long‐term operational stability and functional reliability;^[^ ^16^ ^]^ 2) the inferior stretchability and elasticity of hydrogels impede the device performance improvement and relegate their mechanical robustness.^[^ ^17^ ^]^ Therefore, constructing durable and stretchable hydrogel electronics can enormously promote their practical application in wearable biomonitoring.

Enlightened by human skin, a sophisticated sensing system capable of functioning for over one hundred years due to its regenerative and self‐repairing properties, numerous researchers attempted to impart hydrogel‐based electronics with self‐repairing ability to improve their durability, reliability, and lifespan.^[^ ^18^, ^19^ ^]^ Meanwhile, increasing attempts have been made to enhance the stretchability and elasticity of hydrogel to enhance the device's robustness.^[^ ^20^, ^21^, ^22^ ^]^ Among various hydrogels, Gelatin methacryloyl (GelMA), a skin‐derived polymer, has been extensively used in functional hydrogel construction.^[^ ^23^ ^]^ Due to the abundant cell‐adhesive arginine‐glycine‐aspartic acid (RGD) motifs and matrix metalloproteinase (MMP) cleavage sites, GelMA exhibits remarkable biocompatibility, intrinsic biodegradability, and similar Young's modulus to human tissue, and has emerged as an ideal building block for flexible bioelectronics.^[^ ^24^, ^25^, ^26^, ^27^ ^]^ Despite these advantages, GelMA hydrogels cannot dissipate energy during significant deformations due to the substantial steric hindrance within GelMA chains, resulting in non‐stretchable and fragile characteristics.^[^ ^28^ ^]^ Furthermore, the irreversible nature of the covalent networks in GelMA greatly hinders its self‐healing after damage. The inferior stretchability and self‐healing capability impede the practical usage of GelMA hydrogels in flexible bioelectronics. To address these challenges, researchers have endeavoured to fabricate mechanically reinforced GelMA‐based composite hydrogels with self‐healing capabilities by incorporating non‐covalent interactions (e.g., hydrogen bond (H‐bond),^[^ ^29^, ^30^ ^]^ host‐guest interaction,^[^ ^31^ ^]^ ionic interaction,^[^ ^32^ ^]^ metal‐ligand interaction^[^ ^33^ ^]^) into GelMA networks or integrating dynamic bonds (e.g., schiff base bonds,^[^ ^34^ ^]^ disulfide bond^[^ ^35^ ^]^). For example, Liu et al. reported a GelMA‐based dual‐network hydrogel using tannic acid (TA) as multifunctional H‐bond donors. Compared with the original GelMA hydrogel, GelMA‐TA hydrogel exhibited an elongation and ultimate stress of 225% and 150 kPa, respectively. The healed hydrogel showed no distinct difference in ultimate stresses from its original state.^[^ ^29^ ^]^ Wang et al. developed a self‐healing hydrogel through copolymerization of three‐armed host‐guest supramolecules with GelMA. The resulting hydrogel had an extensibility of ≈70% and a strain self‐healing efficiency of 80%.^[^ ^31^ ^]^ Despite this progress, simultaneous enhancement in both the self‐repairing and mechanical properties of GelMA hydrogels remains an unsolved challenge. The fundamental barrier behind this is their diametrically opposed requirements in chemical bonds. That is, the self‐healing capacity relies on reversible dynamic covalent bonds or noncovalent interactions and high chain mobility, whereas notable mechanical strength necessitates the formation of robust and stable cross‐linking interactions across the polymer chains.^[^ ^36^ ^]^ Moreover, current applications of GelMA hydrogels mainly concentrate on tissue engineering and rarely involve flexible self‐healing sensors.^[^ ^37^ ^]^

In this study, a self‐healing, stretchable, and ionically conductive GelMA‐based hydrogel was successfully fabricated by incorporating polyvinyl alcohol (PVA), N‐(2‐amino‐2‐oxoethyl)‐2‐propenamide (NAGA), sodium tetraborate (borax), and sodium chloride (NaCl) into the GelMA matrix using a two‐step crosslinking methodology. A unique strategy, which combined the interpenetrating networks and multiple interactions involving hierarchical H‐bonds and borate ester bonds (BEBs), was proposed, which effectively balances the conflicting demands of the mechanical and self‐healing properties. Specifically, the constructed interpenetrating networks, strong H‐bonds, and BEBs synergistically improved the mechanical strength, and meanwhile functioned as sacrificial bonds for energy dissipation under deformation. Moreover, the introduced reversible weak H‐bonds and BEBs endowed the hydrogel with the ability to restore its integrity after mechanical damage. Based on this unique design strategy, the resulting GelMA/PNAGA/PVA/borax‐NaCl (GNPB) hydrogel exhibited a remarkable stretchability of ≈160% (fourfold higher), tensile strength of ≈130 kPa (tenfold higher), toughness of 139.1 kJ·m^−3^ (50‐fold higher), and self‐healing efficiency of 86%, compared to pure GelMA hydrogel. Further, a self‐healing GNPB hydrogel strain sensor was developed and exhibited a high gauge factor of ≈3.28, ultra‐low detection limit of 0.1%, and excellent performance recovery capability (e.g., ≈100% in the sensitivity and 75% in the sensing range) after damage. Successful monitoring of both subtle and large‐scale human motions and vital physiological signals using original and healed sensors highlighted the device's durability and longevity in practical usage. This study paved a new avenue for improving the comprehensive performance of GelMA hydrogels and promoted their practical application in wearable bioelectronics.

## 2 Results and Discussion

### 2.1 Synthesis and Characterization of Self‐Healable, Stretchable, and Conductive GNPB Hydrogels

**Figure 1:**
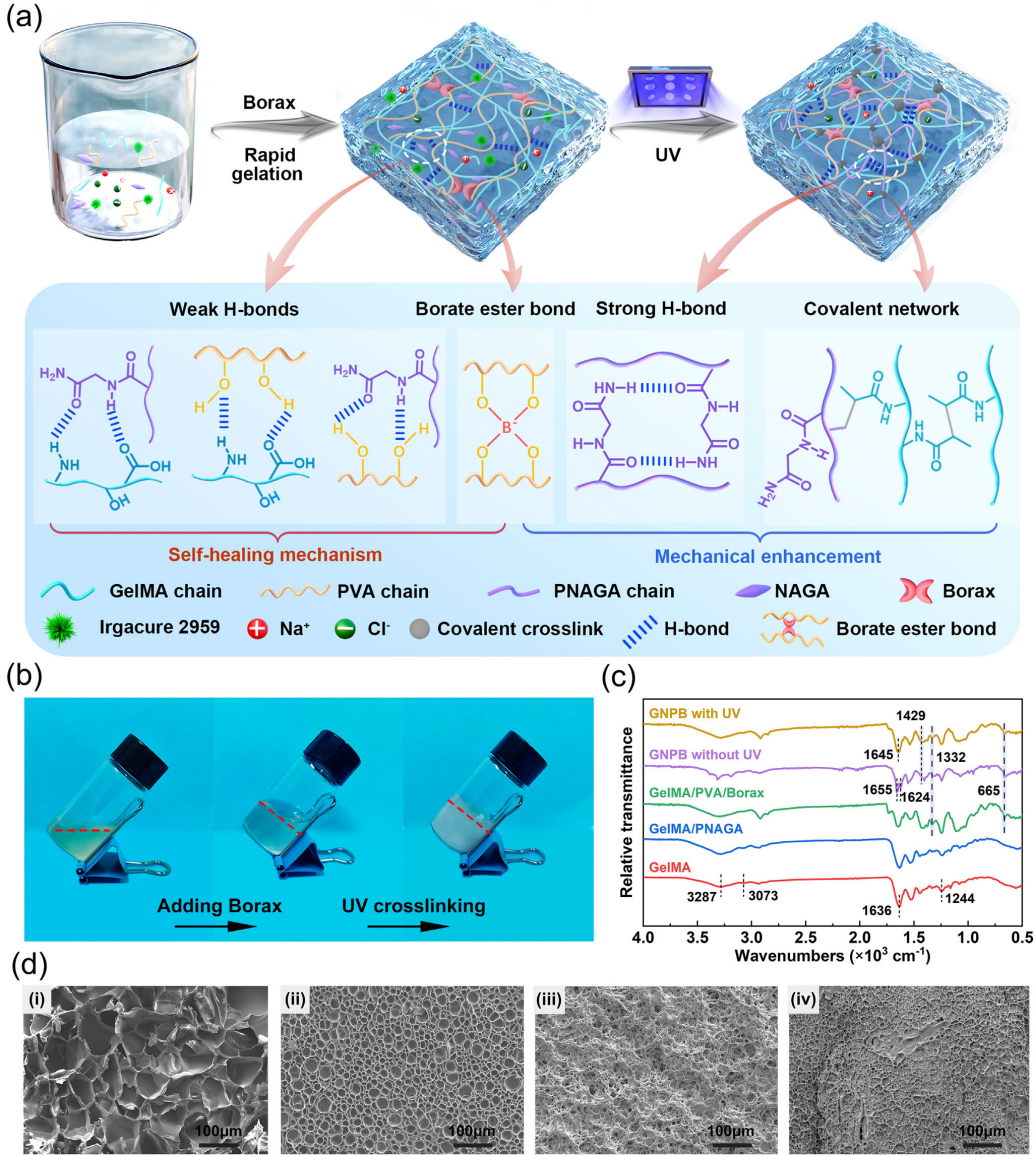
Schematic of the fabrication process and characterization of the GNPB hydrogel. a) Schematic of the fabrication process, internal interactions, and synthesis mechanism. b) Digital photographs of the two‐step crosslinking gelation process of the GNPB hydrogel. c) FTIR spectrum and d) SEM images of i) pure GelMA hydrogel, ii) GN (GelMA/PNAGA) hydrogel, iii) GPB (GelMA/PVA/borax) hydrogel, and iv) GNPB hydrogel.

The preparation process for GNPB hydrogel is demonstrated in **Figure** **1**a,b. In brief, Irgacure 2959, GelMA, NAGA, and NaCl were sequentially dissolved in a PVA solution. Subsequently, a homovolumetric borax solution was introduced to the mixture to generate a pre‐gel, resulting in the rapid creation of BEBs between borax and PVA. The pre‐gel underwent further polymerization via a free radical polymerization reaction under UV irradiation (365 nm, 50 mW·cm^−2^) for five min, forming the GNPB hydrogel. Figure 1b shows the solution transformation states from the mixed solution (before adding borax), to the pre‐gel and final GNPB hydrogel after UV cross‐linking.

The underlying mechanism of the GNPB hydrogel synthesis is illustrated in Figure 1a. Particularly, after the free‐radical polymerisation of NAGA and crosslinking of GelMA, the 3D polymeric framework of the GNPB hydrogel was established through the assembly of GelMA, PVA, and PNAGA chains. The dynamic BEBs were formed via the “di‐diol” complexation of hydroxyl groups (─OH) of PVA with borate ions. The reversible weak H‐bonds among GelMA, PVA, and PNAGA chains and strong dual‐amide H‐bonds between PNAGA chains were formed upon the generation of PNAGA. The interpenetrating polymer networks in the GNPB hydrogel were constructed by the amalgamation of the physical crosslinking networks, dynamic PVA‐borax covalent crosslinking network, and stable covalent crosslinking networks. The increase of crosslinked networks, reinforcement of intermolecular interactions, and strong dual‐amide H‐bonds not only responded to the mechanical strength enhancement but also served to mitigate the entanglement of GelMA chains and improve their mobility. The copious strong and weak H‐bonds and BEBs acted as the sacrificial bonds capable of absorbing the energy necessary for deformation, thereby fortifying the stretchability of the hydrogel. The reversible nature of weak H‐bonds and BEBs imparted the GNPB hydrogel with autonomous self‐repairing capability after injury. These strategies successfully enabled the simultaneous enhancement of the self‐healing and mechanical properties of GelMA hydrogels. Its uniqueness is the ingenious combination of the interpenetrating networks and multiple interactions involving hierarchical H‐bonds and BEBs, which effectively balances the conflicting demands of the mechanical and self‐healing properties. The proposed strategy is universal and can be broadly utilized for biopolymer‐based hydrogels, such as collagen and chitosan, to collaboratively improve their mechanical and self‐healing properties.

The successful synthesis of the GNPB hydrogel was confirmed via chemical structure characterization through Fourier Transform Infrared Spectroscopy (FTIR) and Hydrogen‐Nuclear Magnetic Resonance Spectroscopy (^1^H NMR). As displayed in Figure S1 (Supporting Information), the characteristic signals of the double bond region (5.40 and 5.64 ppm) of methacrylic groups and the reduction in the signal of primary amines of lysine (2.97) on ^1^H NMR spectra of GelMA indicated the successful grafting of MA. Results in Figure 1c indicated that the characteristic peaks of the GelMA hydrogel at 3287, 3073, 1636, and 1244 cm^−1^ corresponding to the O─H/N─H, C─H, C═O, and C─N groups could be observed from the FTIR spectra of all hydrogels.^[^ ^38^ ^]^ Notably, the 1429 and 1332 cm^−1^ peaks within the FTIR spectrum of GNPB hydrogel before UV exposure could be assigned to the asymmetric stretching vibration of B─O─C linkages, and the 665 cm^−1^ peak was associated with the bending vibration of B─O─B linkages. The occurrence of the two linkages signified the establishment of BEBs within the hydrogel network.^[^ ^39^ ^]^ Compared to un‐photopolymerized (no UV exposure) and GNPB hydrogels with UV illumination for 2 min, the absence of the 1624 cm^−1^ peak (C═C bonds) in photopolymerized (5 min of UV exposure) GNPB hydrogels verified the occurrence of free radical polymerization and complete polymerization of the hydrogel (Figure S2, Supporting Information). Additionally, the noticeable red shift of the characteristic peak (C═O groups) from 1655 to 1645 cm^−1^ indicated the formation of robust H‐bonds between PNAGA chains.^[^ ^40^ ^]^

SEM images in Figure 1d‐i–iv showed the microstructures of GelMA hydrogel, GelMA/PNAGA (GN) hydrogel, GelMA/PVA/borax (GPB) hydrogel, and GNPB hydrogel, respectively, which elucidated the internal structural changes under different components. As shown in Figure 1d‐i, the pure GelMA hydrogel displayed prominent interconnected pore networks with substantial pore size. The subsequent introduction of the PNAGA and PVA‐borax system into the GelMA hydrogel led to a notable reduction in pore size due to increased polymerisation degree (Figure 1d‐ii,iii). Notably, the co‐crosslinking of GelMA, PNAGA, and PVA‐borax systems resulted in denser interconnected pores with smaller pore sizes, indicating a highly crosslinked network structure (Figure 1d‐iv). These distinct network and pore size changes reflected macroscopically the improvement in mechanical properties of hydrogels. As illustrated in Figure S3 (Supporting Information), the GNPB hydrogel demonstrates a notable improvement in stretchability and tensile strength compared to the pure GelMA hydrogel. Moreover, in contrast to the GPB hydrogel, the GNPB hydrogel demonstrated a significant alleviation of plastic deformation due to the introduced strong H‐bonds and covalent networks. These results underscored that the proposed strategies, e.g., the introduction of the interpenetrating network structure and multiple interactions including H‐bonds and BEBs, could enable significant improvement of both the stretchability and mechanical toughness of the GelMA hydrogel.

### 2.2 Mechanical Performances of GNPB Hydrogels

**Figure 2:**
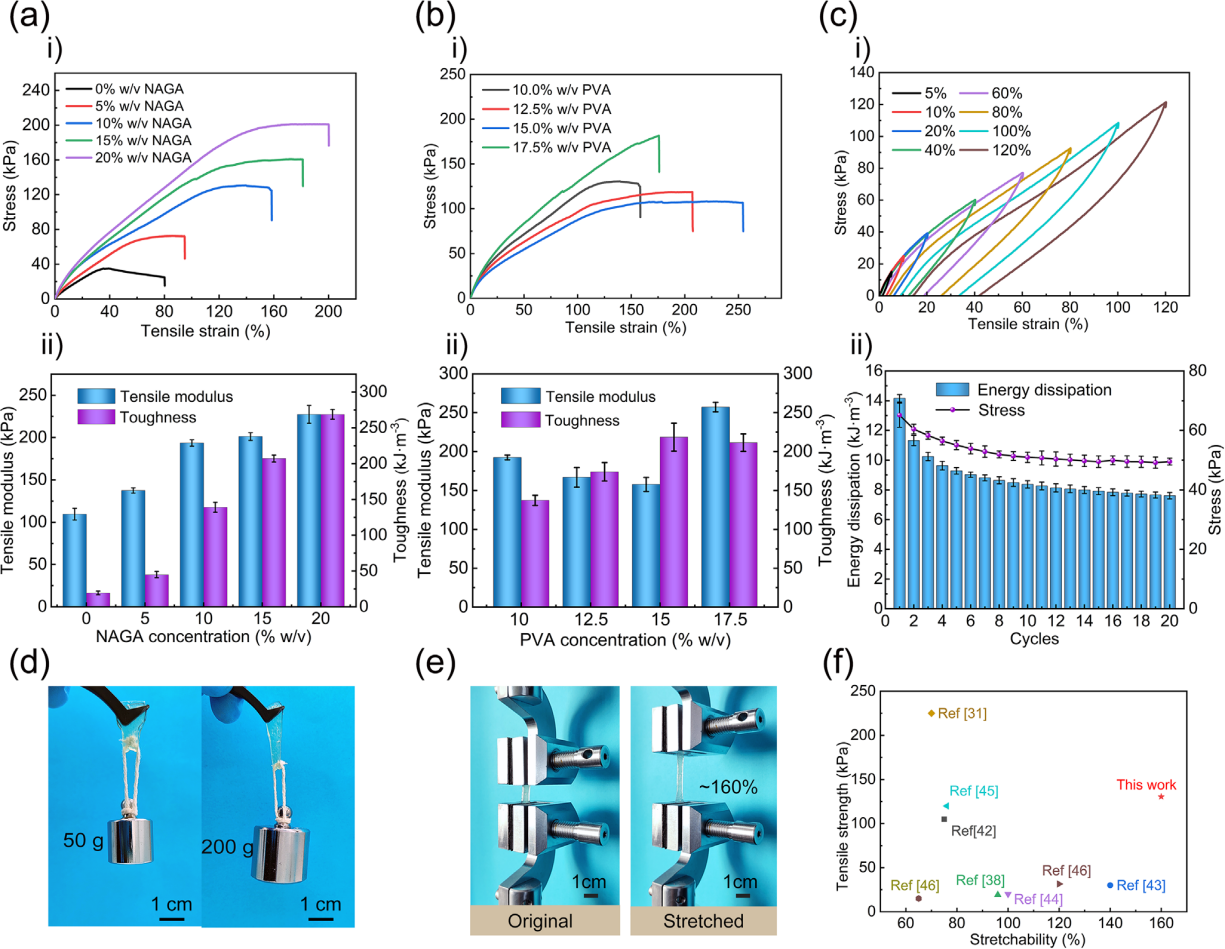
Mechanical performance regulation and characterization of GNPB hydrogel. a) The tensile properties of GNPB hydrogels under different NAGA concentrations. i) Tensile stress‐strain curves. ii) Corresponding tensile modulus and toughness. b) The tensile properties of the GNPB hydrogel under different PVA concentrations. i) Tensile stress‐strain curves. ii) Corresponding modulus and toughness. c) The mechanical properties of the optimal GNPB hydrogel. i) The tensile loading‐unloading tests under various tensile strains. ii) Variation of the energy dissipation and cyclic maximum stress. d) Photographs of GNPB hydrogel holding the weights of 50 and 200 g. e) Demonstration of the stretching test for GNPB hydrogel. f) Comprehensive performance comparison of GNPB hydrogel with GelMA hydrogels reported previously.

The internal interactions and networks play a pivotal role in influencing the mechanical properties of GNPB hydrogels, which can be finely tailored by optimizing the concentration of components, such as NAGA, PVA, and borax. The impact of NAGA concentration was first investigated by varying it from 0 to 20% w/v, while fixing the PVA and borax concentrations at 10% w/v and 1% w/v, respectively. As displayed in **Figures** **2**a and S4 (Supporting Information), the stretchability, strength, tensile modulus, and toughness gradually increased with increasing NAGA concentration. Upon reaching a NAGA concentration of 20% w/v, the stretchability, tensile strength, and toughness achieved their maximum value, that is, ≈200%, ≈0.2 MPa, and ≈0.27 MJ·m^−3^, respectively, exhibiting ≈fivefold, 20‐fold, and 100‐fold enhancement compared to those of the pure GelMA hydrogel. These improvements could be ascribed to the synergistic effects of the covalent network of PNAGA and strong H‐bonds, which significantly enhanced the crosslinking density and reinforced the chain entanglement with GelMA and PVA chains. Further, a systematic investigation was conducted to evaluate the impact of PVA concentration on the mechanical properties of the PVA‐borax system while fixing both GelMA and NAGA concentrations at 10% and the PVA to borax concentration ratio at 10:1.^[^ ^41^ ^]^ The PVA concentration mainly affected the reversible networks formed by weak H‐bonds and BEBs. As shown in Figures 2b and S5 (Supporting Information), an increase in PVA concentration from 10% to 15% w/v led to the improvement of the stretchability and toughness but a decrease in the tensile strength and modulus. Specifically, when the PVA concentration reached 15% w/v, the stretchability of the GNPB hydrogel reached 249.3 ± 15.4%, representing a >sixfold improvement compared to the pure GelMA hydrogel. Notably, under 17.5% w/v PVA concentration, the GNPB hydrogel's strength reached 169.4 ± 11.2 kPa, but the stretchability evidently decreased. That's because the increased crosslinking density at a high PVA concentration resulted in decreased stretchability, while increased stiffness. These results demonstrated that the mechanical properties of the GNPB hydrogel could be highly tailored by adjusting the concentrations of NAGA and PVA.

By combining the aforementioned effects of NAGA and PVA concentrations on mechanical properties, and on self‐healing performance (Section 2.3), the GNPB hydrogel with 10% w/v NAGA, 10% w/v PVA, 1% w/v borax and 1% w/v NaCl was considered as the optimal one, and utilized for further characterization and application. Specifically, the hysteresis behavior and fatigue resistance of the optimal GNPB hydrogel were assessed through cyclic loading‐unloading testing. As depicted in Figure 2c‐i, a notable growth in the hysteresis loops was observed with the strain increasing from 5% to 120%, attributed to the disruption of the crosslinking network for energy dissipation. Additionally, the corresponding energy dissipation ratio remained at ≈40% as the strain increased from 5% to 120%, indicating the favorable and consistent energy dissipation function of the hydrogel networks (Figure S6, Supporting Information). Furthermore, during the 20 consecutive stretches at 50% strain, a distinct hysteresis loop occurred during the initial loading‐unloading cycle, signifying efficient energy dissipation (Figure S7, Supporting Information). The sufficient H‐bonds and BEBs within the hydrogel network served as sacrificial bonds, capable of dissipating substantial energy to withstand deformation until the covalent network was fully compromised. During minor deformation, the internal reversible weak H‐bonds broke first for energy dissipation. As the deformation increased, BEBs played a predominant role in energy dissipation and gradually fractured. Once a certain deformation was reached, the internal strong H‐bonds would also began to contribute to energy dissipation. The broken BEBs were unable to rapidly recover to fully dissipate energy, resulting in a slight residual strain during cyclic loading/unloading. As illustrated in Figure 2c‐ii, the dissipation energy gradually stabilized, and the tensile stress at 50% strain remained at ≈80% of the original stress after 20 cycles, demonstrating commendable recoverability. As illustrated in Figure 2d,e, the optimized GNPB hydrogel could withstand a weight of 200 g and achieved a maximum deformation of approximately 160% in tensile testing. Its elastic modulus was measured at 193.6 ± 3.8 kPa, and its toughness reached 139.1 ± 7.0 kJ·m^−3^, increasing by ≈sevenfold and 50‐fold, respectively, compared with the GelMA hydrogel (with an elastic modulus and toughness of 28.2 kPa and 2.56 kJ·m^−3^, respectively).

In order to confer the hydrogel with the desired conductivity for sensing applications, the inorganic salt NaCl was introduced to construct an ionic conductive pathway, while ensuring the hydrogel's transparency and biocompatibility. To minimize its impact on the mechanical properties of the GNPB hydrogel, the concentration of NaCl was kept below 1% w/v. As shown in Figure S8 (Supporting Information), the strain‐stress curves of the hydrogels with NaCl concentrations varying from 0 to 1% w/v almost overlapped with each other. This result indicated that a low concentration of NaCl had a negligible effect on the mechanical properties of the hydrogel. In this content, the concentration of NaCl (ranging from 0–1% w/v) was further optimized based on its impact on the conductivity and strain sensitivity. As shown in Figure S9a (Supporting Information), the hydrogel's conductivity increased from 0.2 to 0.6 S·m^−1^ with the NaCl concentration increasing from 0 to 1% w/v. Furthermore, the strain sensitivity improved from 1.64 to 1.98 within 80% strain and from 2.35 to 2.82 in the strain range of 80–160%, which was sensitive enough for various human motions detection (demonstrated in Section 2.5) (Figure S9b, Supporting Information). Importantly, a 1.0% NaCl concentration closely matched physiological saline levels (0.9% NaCl), minimizing osmotic pressure effects and enhancing biocompatibility for wearable sensor applications. Therefore, 1.0% w/v was selected as the concentration of NaCl for the proposed GNPB hydrogel.

Moreover, we conducted a comparative analysis of the mechanical properties of the optimal GNPB hydrogel with various GelMA hydrogels developed previously,^[^ ^31^, ^38^, ^42^, ^43^, ^44^, ^45^, ^46^, ^47^ ^]^ encompassing aspects such as stretchability and tensile strength. As shown in Figure 2f, the GNPB hydrogel exhibited an exceptional stretchability of 160% and a notable tensile strength of approximately 130 kPa, demonstrating fourfold and tenfold improvement in stretchability and tensile strength, respectively, compared to those reported previously. These results demonstrated that the introduction of interpenetrating polymer networks, strong H‐bonds, and BEBs into the GelMA network can effectively reinforce the mechanical properties of GelMA.

### 2.3 Self‐Healing Performances of GNPB Hydrogels

**Figure 3:**
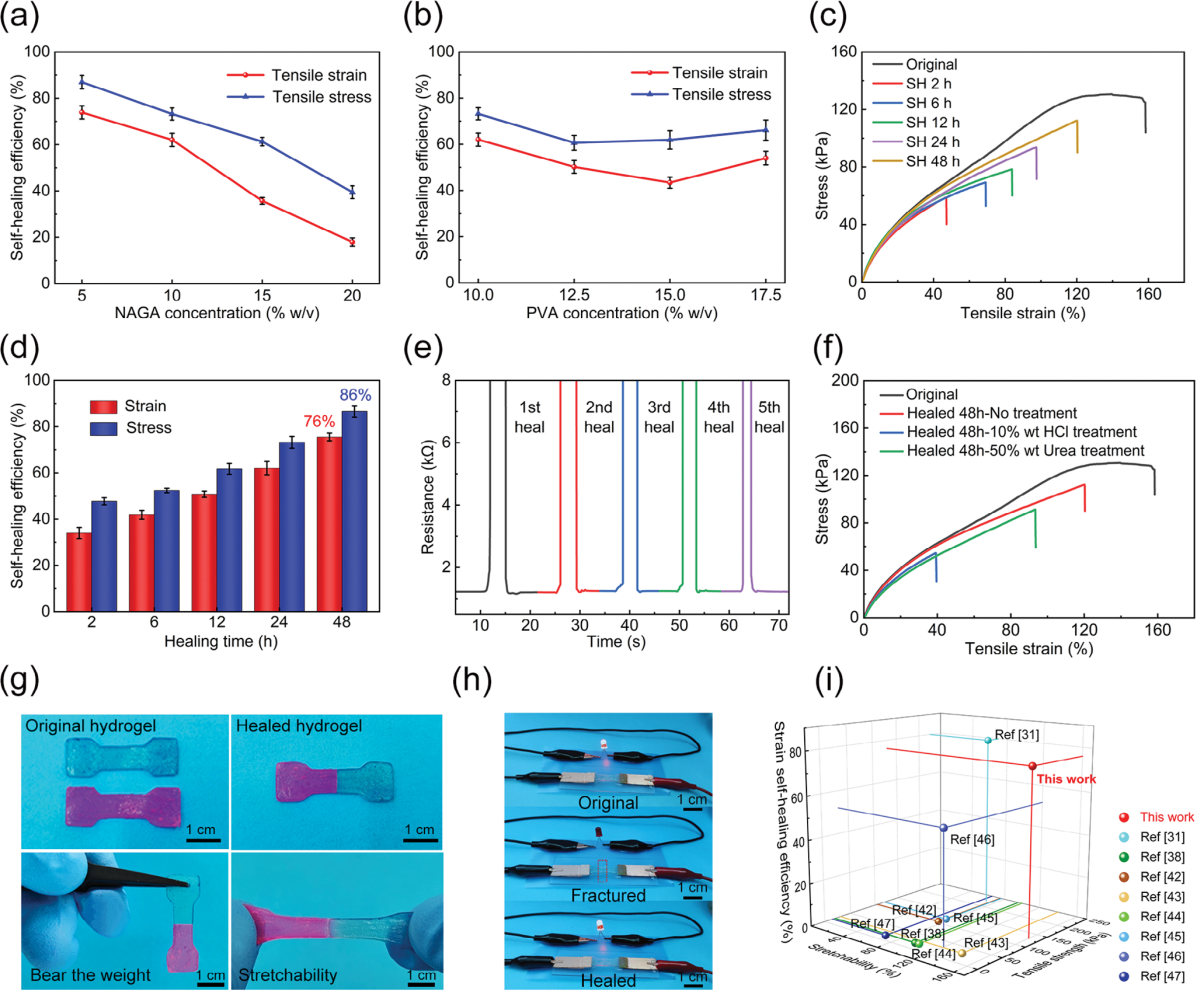
Self‐healing properties characterization of GNPB hydrogel. a) The tensile strain and stress SHEs at different NAGA concentrations. b) The tensile strain and stress SHEs at different PVA concentrations. c) Tensile stress–strain curves of GNPB hydrogels under different healing times at 25 °C. d) The corresponding strain and stress SHEs. e) Real‐time resistance of hydrogel during 5 fractured‐healed cycles. f) Stress–strain curves of the original and healed hydrogels after treating the fractured surface with different solutions. g) Macroscopic mechanical self‐healing display, h) Conductivity repairing display using the hydrogel as a connector. i) A comprehensive comparison of stretchability, tensile strength, and self‐healing of our GNPB hydrogel with those reported previously.

The reversible network comprising weak H‐bonds and dynamic BEBs not only endowed GNPB hydrogel with outstanding mechanical properties but also contributed to remarkable self‐healing capabilities. Herein, the impact of NAGA and PVA concentrations on tensile self‐healing efficiency (SHE) was investigated under healing at 25 °C for 24 h. As illustrated in **Figure** **3**a, it is noteworthy that both the strain and stress SHEs exhibited a significant decrease as the NAGA concentration increased from 5% to 20% w/v. Specifically, the SHEs in fracture strain and maximum strength decreased from 73.9 ± 2.8% and 86.9 ± 2.9% to 18.0 ± 1.7% and 39.5 ± 2.8%, respectively. The underlying reason was that an increase in NAGA concentration introduced more covalent networks and irreversible dual‐amide H‐bonds, thereby reducing dynamic and reversible interactions and consequently restraining the SHEs. Figure 3b shows the effect of PVA concentration on the strain and stress SHEs. The strain SHE decreased from 62.1 ± 2.9% to 43.4 ± 2.4% with the PVA concentration changing from 10% to 15% w/v. The stress SHE of the hydrogel decreased from 73.2 ± 2.6% to 60.7 ± 3.2% as the PVA concentration changed from 10% to 12.5%, and with a continued ascending of the PVA concentration to 17.5%, the stress SHE rose to 66.1 ± 4.4%. The relatively robust self‐repairing ability after 24 h of healing was due to the abundant presence of BEBs. Based on these regulations, both the PVA and NAGA concentrations were determined as 10% w/v to make a trade‐off between mechanical and self‐healing properties.

The self‐healing ability of the optimized GNPB hydrogel was further validated through microscopic morphology repair and sectional structure restoration. First, Laser Scanning Confocal Microscopy (LSCM) was used to characterize the healed interface. As depicted in Figure S10 (Supporting Information), the cracked interface was gradually repaired, resulting in a smooth morphology with a faint fractured scar after 30 min of healing. Additionally, the healing process was visually observed via SEM, revealing the restoration of the interfacial polymer network. As illustrated in Figure S11a (Supporting Information), the cracked hydrogel sample displayed a reconnected yet misaligned interface, indicating the reformation of polymer networks bridging the fractured segments. Subsequently, the healed hydrogel (Figure S11b, Supporting Information) demonstrated a more pronounced repaired effect, with the scars of some healed regions being nearly imperceptible (Figure S11b‐iii, Supporting Information), providing visual evidence of the hydrogel's resurfacing capabilities.

To comprehensively evaluate the self‐healing capabilities, the mechanical and electrically self‐healing properties of the optimal GNPB hydrogel were further experimentally characterized. Figure 3c illustrates the tensile stress‐strain curves of the rejoined hydrogels that were measured after healing for 2, 6, 12, 24, and 48 h in a wet and enclosed environment at 25 °C. Both the fracture strain and stress increased with the increasing healing time. For instance, the GNPB hydrogel exhibited fracture strain and stress of 54.4% and 62.3 kPa after healing for 2 h, respectively, which corresponded to the strain and stress SHEs of 34.0% and 47.7%. When the healing time increased to 48 h, the fracture strain and stress significantly increased to ≈120% and 112 kPa, respectively, approximating those of the original hydrogel. The corresponding strain and stress SHEs increased 76.0 ± 1.7% and 86.4 ± 2.5% after 48 h of healing, suggesting superior mechanical repair capability (Figure 3d). This was because the healing time affects the polymer chain diffusion to re‐form dynamically reversible H‐bonds and BEBs. The longer time could result in more reformed interfacial interactions and reconstructed networks. As illustrated in Figure 3g and Movie S1, two identical hydrogels were stained using Rhodamine B and methyl green, respectively. Subsequently, two segments of the cracked hydrogels with different colors were seamlessly rejoined with gentle pressure. The reconnected hydrogel could support its weight after merely 1 min of healing and maintain a degree of stretchability after 1 h of healing, underscoring its rapid mechanical properties restoration. Ultimately, the cut/healed hydrogel regained ≈120% of its stretchability after 48 h of healing. Moreover, the electrical self‐healing capability was further exemplified by an on‐off LED circuit, wherein the conductive pathway was swiftly reestablished, leading to the illumination of the LED (Figure 3h). The resistance of the hydrogel exhibited consistent recovery throughout the 5 fractured/healed cycles (Figure 3e). As depicted in Figure S13 (Supporting Information), the conductive pathway could be reinstated in <1 s, and the electrical conductivity could return to its original state within 3.5 s, demonstrating the exceptional electrical self‐repairing capability of the GNPB hydrogel.

In addition, to evaluate the impact of the temperature on the SHE of GNPB‐hydrogels, we experimentally tested the strain–stress curves under different temperatures. The results were shown in Figure S12 (Supporting Information). It could be seen that both the fracture stress and strain of the tested hydrogel samples increased with the elevated temperatures under the same healing time of 2 h. The mechanism behind this is that the thermally responsive nature of H‐bonds could accelerate their dynamic reconfiguration in hydrogels at elevated temperatures, thus improving the SHE.

To further elucidate the primary self‐healing mechanism, the tensile‐stress curves of the healed hydrogel were tested subsequent to treating the fractured interfaces with different solutions. Specifically, the HCl and urea solutions were employed to disrupt the BEBs and H‐bonds in the bisected interface of two GNPB hydrogel samples, respectively. The tensile‐stress curves of the two treated hydrogel samples were tested after healing for 48 h at 25 °C, and compared to the GNPB hydrogel sample without any treatment. The results showed that the healed hydrogel samples treated with 50% wt urea achieved strain and stress SHEs of 59.4 ± 3.2% and 69.4 ± 1.8%, respectively, while those of the samples treated with a 10% wt HCl solution reached only 26.0 ± 2.7% and 39.2 ± 1.6%. (Figure 3f; Figure S14, Supporting Information). In comparison, both treated hydrogel samples exhibited much lower SHEs than those of the GNPB hydrogels without any treatment (strain and stress SHEs are 76.0 ± 1.7% and 86.4 ± 2.5%), which meant that the disruption of the BEBs or H‐bonds could significantly decrease the SHE. These results demonstrated that both BEBs and H‐bonds were responsible for the self‐healing properties of the GNPB hydrogel, and the BEBs contributed more to the SHE. Meanwhile, it also indicated that the synergistic effect of BEBs and H‐bonds contributed to the self‐repairing process and excellent SHE of the GNPB hydrogel.

We further compared the mechanical and self‐healing properties of GNPB hydrogels with those of GelMA hydrogels reported previously.^[^ ^31^, ^38^, ^42^, ^43^, ^44^, ^45^, ^46^, ^47^ ^]^ As displayed in Figure 3i and Table S1 (Supporting Information), besides its outstanding mechanical properties, our hydrogel also had an impressive tensile strain SHE of 76%, demonstrating excellent comprehensive performance. These results indicated the feasibility of integrating interpenetrating networks and incorporating hierarchical H‐bonds and BEBs into GelMA hydrogels to achieve synergistic enhancements in mechanical and self‐healing properties. In addition, to demonstrate the biocompatibility of GNPB hydrogel, mouse fibroblast (L929) cells were cocultured with different concentrations of hydrogel extracts, and the viability of the cells was detected by Cell Counting Kit‐8 (CCK‐8) assay. As shown in Figure S15 (Supporting Information), the cell viability remained above 95% after 3 days of incubation in all tested groups, indicating the excellent biocompatibility of the GNPB hydrogel.

### 2.4 GNPB Hydrogels‐Based Wearable Self‐Healing Strain Sensors

**Figure 4:**
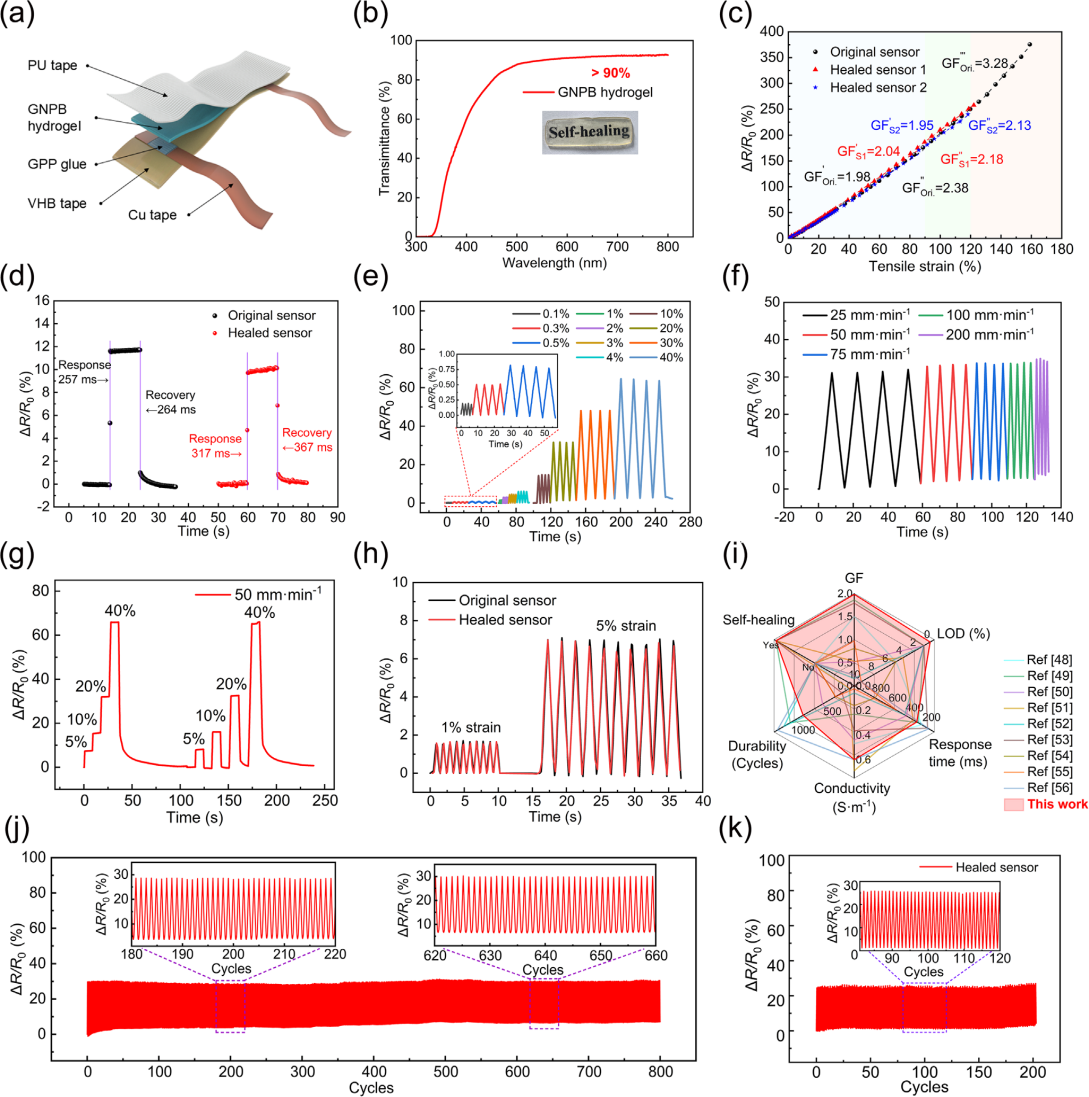
The structure and sensing performances of the GNPB hydrogel‐based self‐healing strain sensors. a) Structure schematic of the strain sensor. b) Transparency of the GNPB hydrogel. c) Gauge factor of the self‐healing strain sensor. d) The response/recovery time of the original and healed sensors tested via an instant stretching‐releasing at 10% strain. e) Δ*R*/*R*_0_ curves under cyclic stretching‐releasing under various strains. f) Dynamic responses under testing speeds from 25–200 mm·min^−1^ at the strain of 20%. g) Δ*R*/*R*_0_ curves responding to the stepwise strain (5–40%). h) Δ*R*/*R*_0_ curves of the original and healed sensors at 1% and 5% strain. i) Comprehensive comparison of sensitivity, self‐healing ability, LOD, response time, conductivity, and durability. j) Durability tests of the original sensor under 20% strain for 800 cycles. k) Durability tests of the healed sensor under 20% strain for 200 cycles.

Encouraged by their impressive stretchability, self‐healability, conductivity, and exceptional strain‐induced resistance response, we further explored the applications of GNPB hydrogels in self‐healing tactile sensors. As illustrated in **Figure** **4**a, the strain sensor was constructed with VHB tape as the adhesive substrate, GNPB hydrogel as the sensing layer, medical PU tape as the encapsulation layer, Gelatin/Polyacrylic acid/Poly[2‐(methacryloyl‐oxy)ethyl]dimethyl‐(3‐sulfopropyl) ammonium hydroxide (GPP) hydrogel glue as the intermediate adhesive layer, and copper tape as the wire. During its fabrication, the GPP hydrogel glue was uniformly applied at the interface between the GNPB hydrogel and copper foil, where it underwent in situ polymerisation with the GNPB hydrogel. The formed covalent networks and H‐bonds between the GNPB hydrogel and GPP adhesive, along with the electrostatic interactions between the Cu tape and GPP adhesive, enabled a strong adhesion of the hydrogel to the Cu tape, ensuring the device stability and preventing detachment or slippage during deformation (Figure S16, Supporting Information). A shear test was conducted for the adhesive strength evaluation, revealing a maximum shear stress of ≈27 kPa (Figure S17a, Supporting Information). The presence of residue on the surface of the Cu tape indicated the robust bonding capability between the interfaces (Figure S17b, Supporting Information).

The digital images illustrated the excellent transparency and flexibility of the developed sensor (Figure S18a, Supporting Information). The transparency of the GNPB hydrogel sensing layer and the sensor were further characterized using UV–vis spectroscopy (Figure 4b; Figure S18b, Supporting Information). The result revealed that the hydrogel possessed a transmittance of ＞90% across the wavelength range of 500–800 nm, and the whole sensor maintained a transmittance exceeding 80%, rendering it great promise in invisible wearable technology. The strain sensitivity (GF) of the sensor was ascertained by calculating the slope of the linear fitting of the relative resistance change curves over the tensile strain range of 0–160%. The original sensor displayed a superior GF of 1.98 within 80% strain, escalating to 2.38 between 80% and 120% strain, and reaching a peak GF of 3.28 within the 120–160% strain range. Remarkably, the healed sensor nearly fully restored the original sensitivity within the 80% strain range (Figure 4c). Additionally, the healed sensor exhibited a detectable strain range of 120%, recovering ≈75% of the detection range of the original sensor. Figure 4d shows the response/recovery time of the original and healed sensors. The original sensor demonstrated a response time of 257 ms at 10% strain under a testing speed of 200 mm·min^−1^ utilizing a tensile tester. In comparison, the healed sensor exhibited a slightly longer response time of 317 ms. The slightly prolonged response time was probably caused by the partial non‐repair of the conductive network.

Furthermore, the sensors demonstrated reproducible and stable responses when subjected to varying strains (0.1–40%) (Figure 4e) and testing speeds (25–200 mm·min^−1^) (Figure 4f), underscoring the sensor's robust and reproducible performance in detecting various signals. Moreover, the sensor exhibited the capability to detect minor deformations with an ultra‐low limit of detection (LOD) of 0.1%, rendering its ability to monitor subtle human movements. Results in Figure 4g further manifested the sensor's commendable dynamic response and stability. A comparison between the original and healed sensors in detecting 1% and 5% strain signals showcased the sensor's excellent sensing self‐healing ability (Figure 4h). Additionally, the sensor's resolution was assessed by detecting the Δ*R*/*R*_0_ curves from 5% to 5.1% strain, revealing an impressive resolution of 0.1% strain (Figure S19, Supporting Information). Durability, a crucial aspect of flexible wearable sensors, was evaluated through 800 continuous loading‐unloading cycles under 20% strain. The results demonstrated that the original sensor exhibited a high durability of 800 cycles (Figure 4j). In comparison, the healed sensor maintained durability for 200 cycles with only a slight decrease in the detected peak value, which was primarily attributed to the partial loss of water in the hydrogel's networks (Figure 4k). The slight variation in the relative resistance at the beginning of the cyclic testing could be ascribed to the micro‐level chain segment slippage and fatigue fracture within the hydrogel network, which could lead to a minor permanent structural deformation, and the unstable contact between the sensor and the force loading head of the used testing machine. However, after a certain number of training cycles, the sensor reached a relatively stable operating state with high durability. Stability testing of the sensor at a 20% tensile strain over 4 h was presented in Figure S20 (Supporting Information). The basically unchanged relative resistance after testing for 4 h confirmed its robust stability over a short duration.

Compared with other reported natural polymer hydrogel‐based strain sensors,^[^ ^48^, ^49^, ^50^, ^51^, ^52^, ^53^, ^54^, ^55^, ^56^ ^]^ our GNPB hydrogel‐based sensor boasted high sensitivity within 80% strain, an ultra‐low LOD (0.1%), relatively rapid response time, high conductivity, and good durability, particularly their outstanding sensing self‐healing capability (Figure 4i; Table S2, Supporting Information). These advancements highlighted the tremendous promise of our unique structural design approach in advancing the reliability, durability, and longevity of natural polymer‐based hydrogel flexible sensors for demanding engineering applications.

### 2.5 Applications in Wearable Medical Monitoring

**Figure 5:**
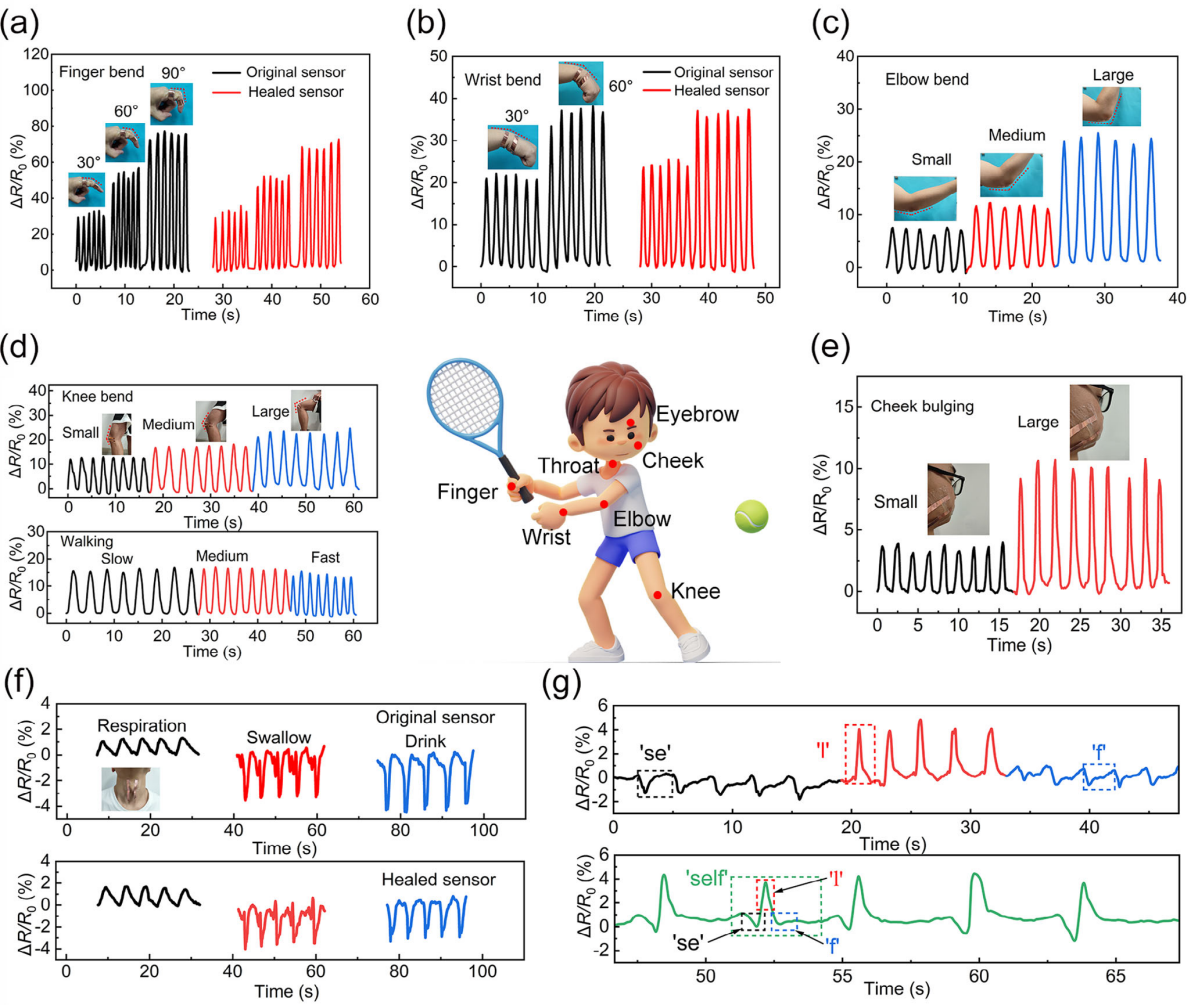
The original and healed hydrogel strain sensors for monitoring various human motions in real‐time. a) Finger bending, b) wrist bending, monitoring by the original and healed sensors. c) Elbow bending, d) knee bending, and walking speed, e) cheek bulging of the original sensor. f) The original and healed sensors are attached to the throat for respiration, swallowing, and drinking detection. g) Pronouncing detection for letter/word recognition.

Benefiting from the strong adhesive property of the VHB tape substrate and the matched modulus to human skin, the GNPB hydrogel‐based self‐healing strain sensor could be conformally adhered to diverse regions of the human body, facilitating the acquisition and analysis of vital physiological signals. Furthermore, its remarkable sensing self‐healing capability enhanced its resilience to physical damage, ensuring prolonged, reliable, and secure medical monitoring. To validate these unique merits, the strain sensor was first attached to various human joints, such as finger, wrist, elbow, and knee. As displayed in **Figure** **5**a and Movie S2 (Supporting Information), both the original and healed GNPB hydrogel strain sensors exhibited a distinct increase in the Δ*R*/*R*_0_ when the volunteer's finger bent from 0 to 30°, 60°, and 90°. For a given bending angle, both sensors output consistent peaks and baselines in the Δ*R*/*R*_0_ under multiple repeated bending. Importantly, the healed sensor with 1 h healing time in situ could produce almost the same Δ*R*/*R*_0_ changes (with a small relative deviation of 8% at a bending angle of 90°) as those of the original sensors before cutting. These results demonstrated that our GNPB hydrogel‐based self‐healing strain sensor could accurately discern the finger's bending angles and almost completely recover its sensing capability after damage. Similarly, when monitoring the wrist bending motion, the original and healed sensors showed highly identical response curves under the bending angles of 30°and 60°. For instance, the Δ*R*/*R*_0_ changes for the original and healed sensors were 36.58% and 36.50% at 60°, respectively, with a relative deviation as small as 0.2%. This further validated the excellent performance recovery capability and lifespan of our self‐healing strain sensor (Figure 5b). Furthermore, the strain sensor successfully captured substantial motion amplitudes from the elbow, knee, and neck (Figure 5c,d; Figure S21, Supporting Information), as well as dynamic motions like walking with different moving frequencies. For all these tests, distinct and stable changes in the Δ*R*/*R*_0_ were presented, demonstrating the excellent capability of the developed GNPB hydrogel‐based self‐healing strain sensor in monitoring human joint motions with various motion amplitudes and speeds.

Owing to its exceptional sensitivity, low LOD, and enduring stability, the sensor was also capable of detecting subtle deformations, such as cheek bulging and throat vibration. As shown in Figure 5e, the sensor affixed to the cheek could successfully measure the cheek bugling, with distinguishable Δ*R*/*R*_0_ variations are 3.58% and 9.96% under small and large bugling amplitudes. Moreover, when placed on the throat, the sensor was able to identify static respiration, swallowing, and drinking, with relative resistance variations of 1%, 3.5%, and 4%, respectively (Figure 5f). This was reasonable because the throat skin strains induced by the respiration, swallowing, and drinking increased sequentially. Importantly, the healed sensor produced stable signals with closely identical waveforms as those of the original sensor under the same motions, indicating its excellent self‐healing capability. Besides, the GNPB hydrogel‐based self‐healing strain sensor was also adhered to the volunteer's wrist to monitor human pulse and heart rate variations. Both the original and healed sensors exhibited continuous pulse waveforms (Figure S22, Supporting Information). Especially, the two key peaks, including the impulse (P_1_) and tidal (P_2_) waves, could be obviously observed from the two pulse waveforms, and the heart rates calculated from the waveforms were both 80 bpm. These results demonstrated the outstanding sensitivity and repairing ability of our self‐healing strain sensor. Furthermore, it could generate distinguishable responses to carious spoken letters or words, including “stretchable”, “hydrogel”, “Ni hao”, and “Xi'an” (Figure S23, Supporting Information). Notably, as shown in Figure 5g, the pronunciation of the word “self” aligned with the phonetic breakdown of its constituent letters (“se”, “l”, and “f”), demonstrating its potential for speech recognition applications. These experimental results indicated that the GNPB hydrogel‐based self‐healing strain sensors offer substantial promise for the advancement of next‐generation wearable medical devices in the realm of public health.

## 3 Conclusion

In conclusion, we have successfully developed a highly stretchable, self‐healing, and conductive GelMA hydrogel by introducing PVA, NAGA, borax, and NaCl into the GelMA polymer networks via a two‐step polymerization process. The underlying mechanism relies on the combination of the interpenetrating networks and multiple interactions involving hierarchical H‐bonds and BEBs, which effectively balances the conflicting demands of the mechanical and seal‐healing properties. The weak H‐bonds and BEBs imparted the hydrogel with self‐healing ability, while the synergistic effect of the strong H‐bonds, BEBs, and interpenetrating networks contributed to both the mechanical strength and stretchability enhancement. Experimental testing demonstrated remarkable stretchability (160%), tensile strength (≈130 kPa), and self‐healing efficiency (86%) of the developed hydrogel, validating the feasibility of the design strategy. Furthermore, a GelMA hydrogel‐based self‐healing strain sensor was fabricated, which demonstrated exceptional performances, including a high gauge factor (≈3.28), ultra‐low detection limit (0.1%), superior durability, and nearly full recovery of sensing performance after damage. These advantages enabled both the original and healed sensors to monitor a wide range of human body motions. This research opened new avenues for improving the comprehensive performance of GelMA hydrogels and accelerated their application in wearable bioelectronics. Future work can focus on the optimization of the NaCl concentration and the ratio of PVA to borax to improve the strain sensitivity and self‐healing performance, respectively, as well as the performance under extreme environmental conditions such as low temperatures and high humidity. In addition, the proposed unique strategy for synthetically improving the mechanical and self‐healing properties can also be broadly utilized for biopolymer‐based hydrogels, such as collagen and chitosan, due to their similar functional groups to GelMA hydrogel.

## 4 Experimental Section

### Materials

Gelatin from porcine skin (Type A, gel strength ≈300 g Bloom), methacrylic anhydride (MA, 94%), and Irgacure 2959 photoinitiator (98%) were acquired from Sigma Aldrich. Polyvinyl Alcohol (PVA, degree of alcoholysis, 86.5–89.0 mol%), sodium tetraborate (borax, ≥ 99%), sodium chloride (NaCl, 99.5%), urea (99%), HCl (AR), [2‐(Methacryloyloxy)ethyl]dimethyl‐(3‐sulfopropyl) ammonium hydroxide (SBMA, ≥ 97%), methyl green (AR), and Rhodamine B (AR) were acquired from Aladdin. N‐(2‐Amino‐2‐oxoethyl)‐2‐propenamide (NAGA, ≥ 97.0%) was supplied by Shanghai Shenze Chemical Technology Co., LTD. Acrylic acid (AA, 98%) was procured from Meryer. Dulbecco's Phosphate Buffered Saline (DPBS, without Ca^2+^ and Mg^2+^) was provided by Xi'an Haimeng Experimental Technology Co., LTD. Deionized water (DI water, 18.2 MΩ) from the laboratory.

### Preparation of GelMA/PNAGA/PVA/borax‐NaCl Hydrogels

The as‐obtained GNPB hydrogel was synthesized through a two‐step crosslinking approach, utilizing borax as the cross‐linker and UV light to initiate free radical polymerization further. Briefly, a 20% w/v PVA solution was obtained by dissolving 1 g of PVA into 5 mL of DI water at 90 °C for 3 h. Then added 0.1 g of Irgacure 2959, 0.1 g of NaCl, and 1 g of NAGA into the PVA solution, which was heated at 80 °C with constant magnetic stirring until a clear and uniform mixture was achieved. After that, 1 g of GelMA was solubilized in the mixed solution at 50 °C, and the resultant solution was further centrifuged to eliminate bubbles. Subsequently, 5 mL of 2% w/v borax solution was added into the resulting mixture, and the prepared pre‐gel was homogenized by mechanical stirring and centrifugal oscillation. Finally, the pre‐gel was shaped using a dumbbell PDMS mold and further polymerized under UV light (DYMAX BlueWave@200, USA) at 50 mW·cm^−2^ for 5 min to obtain the GelMA/PNAGA/PVA/borax‐NaCl (denoted as GNPB hydrogel). In particular, the mass concentration ratio of PVA and borax is maintained at 10: 1. Additionally, a pure GelMA hydrogel was prepared using 10% w/v GelMA and 1% w/v Irgacure 2959 photoinitiator.

### Hydrogel Characterization

^1^H NMR spectra of gelatin and GelMA were recorded on a nuclear magnetic resonance hydrogen spectrometer (Bruker AVANCE 400). Fourier transform infrared (FTIR) spectra of GelMA, GN, GPB, GNPB without UV irradiation, and GNPB hydrogel with UV irradiation were recorded on a micro‐infrared spectrometer (Bruker VERTEX70, Germany) in the range of 4000–500 cm^−1^. The SEM images of pure GelMA, GN, GPB, and GNPB hydrogel were taken with a scanning electron microscope (Hitachi SU‐8010, Japan). The transmittance was tested by an ultraviolet‐visible spectrophotometer (Shimadzu UV‐3600, Japan).

### Mechanical and Self‐Healing Property Measurement

The mechanical property measurements were carried out on a universal testing system (SUST CMT1202, China) incorporating a 200 N load cell. The measured sample was shaped into a dumbbell with dimensions of 15 mm in length, 7 mm in width, and 2 mm in height. The test was carried out at room temperature (25 °C) and the tensile test speed was set at 50 mm·min^−1^. The tensile elastic modulus (E) was defined as the fit slope of the tensile stress–strain curves (0–20% strain). The calculation methods of the toughness (Γ), energy dissipation (ΔUi), and energy dissipated ratio (δ) were given in the Supporting Information. The adhesive strength between the GNPB hydrogel and Cu tape was measured by lap‐shear testing. All the tests were conducted at least 3 times for each group.

The surface morphology characteristics of the healed interface were observed under a laser scanning confocal microscope (LSCM, Olympus OLS4000, Japan). The rejoining of the damaged crack was characterized using SEM (Hitachi SU‐8010, Japan). The optical images of the cut/healed hydrogel were recorded by an optical microscope (Nikon SMZ‐2, Japan). The on‐off LED circuit was powered by a digital source meter (Keithley 2602 B, USA) at a voltage of 9 V. The measured sample was molded into a dumbbell shape with dimensions of 15 mm in length, 7 mm in width, and 2 mm in height. The self‐healing properties were visually characterized by cutting the hydrogel into two pieces, staining, reconnecting, and keeping it in wet and enclosed conditions for 48 h, and then characterized qualitatively and quantitatively.

### The Assembly and Performance Testing of the Strain Sensor

First, two Cu foil tapes were affixed to the two sides of the VHB double‐sided tape as the conductive wires. A coating of the gelatin/AA/SBMA precursor solution was applied to the surface of copper (Cu) tapes, serving as an adhesive layer. After that, the stretchable PU tape was placed on top as the encapsulation layer. Finally, the integrity was exposed to UV light for in‐situ polymerization to bond the GNPB hydrogel and Cu tape. Figure S24 (Supporting Information) presents the testing system for the GNPB hydrogel‐based strain sensor. After connecting the strain sensor to the precision LCR meter (KEYSIGHT E4980A, USA), the relative resistance changes with strain were tested at 1 kHz with a 1 V AC signal. The diverse human motion signals were examined by attaching the original and healed sensor to the skin of volunteers.

### Biocompatibility Evaluation

L929 cells were chosen to evaluate the cytocompatibility of GNPB hydrogel by CCK‐8 assay. First, cells were cultured in Dulbecco's Modified Eagle Medium (DMEM), supplemented with 10% fetal bovine serum and 1% penicillin‐streptomycin. Briefly, cells were seeded in 96‐well plates at a density of 6000 cells per well and incubated at 37 °C and 5% CO_2_ for 24 h. Then the culture medium was replaced with a fresh medium that contained extracts of different concentrations of GNPB hydrogel (1, 5, and 10 mg mL^−1^). L929 cells treated without hydrogel extracts were used as the control. After being co‐cultured for 1 day or 3 days, the culture medium was removed, and cells were washed twice with PBS. Then 100 µL of CCK‐8 (10% v/v) working solution was added to each well, and cells were further incubated for 1 h. The absorbance of each well at 450 nm was measured using a microplate reader (TECAN, Infinite 200 PRO) to calculate the cell viability.

### Peking University Third Hospital Approval for Human Subject Testing

The conducted human subject experiments were performed in compliance with the protocols that have been approved by the Medical Science Research Ethics Committee of Peking University Third Hospital (IRB00006761‐M2024893). All subjects gave written informed consent before participation in the study. For all demonstrations on human skin, signed consent was obtained from the volunteer.

### Statistical Analyses

Statistical analysis was performed with Origin 2021. All experiments were repeated parallelly at least three times and the experimental data were presented as the mean ± SD.

## Conflict of Interest

The authors declare no conflict of interest.

## Author Contributions

Z.K.L. conceptualized the study, provided guidance for the methodology, proofread the data, and revised the manuscript. B.W. and J.J.L. conducted the experiments, fabricated and characterized the device, analyzed the experimental data, and drafted the original manuscript. Y.M.X. provided guidance for the methodology and revised the manuscript. J.X.W., B.Q.J., M.A.K.Q., L.Y., and K.Z. were in charge of the manuscript formatting, expressions, and references. G.Y.H. provided guidance for the methodology on the human body experiment and validated the data. Y.H.Z. and M. Li revised the manuscript. P.Y. and D.J.L. proofread the data and formatted the pictures and tables. Z.K.L. and L.B.Z. were in charge of supervision, project administration, and funding acquisition.

## Supporting information



Supporting Information

Supplemental Movie 1

Supplemental Movie 2

## Data Availability

The data that support the findings of this study are available from the corresponding author upon reasonable request.

## References

[1] Y. Ye , F. Jiang , Nano Energy 2022, 99, 107374.

[2] X. Liu , J. Liu , S. Lin , X. Zhao , Mate. Today 2020, 36, 102.

[3] X. Zhang , X. Chen , Z. Ye , W. Liu , X. Liu , X. Wang , J. Mater. Chem. C 2023, 11, 10785.

[4] S. Y. Zheng , S. Mao , J. Yuan , S. Wang , X. He , X. Zhang , C. Du , D. Zhang , Z. L. Wu , J. Yang , Chem. Mater. 2021, 33, 8418.

[5] L. Zhao , Z. J. Ren , X. Liu , Q. J. Ling , Z. J. Li , H. B. Gu , A. Multifunctional , ACS Appl. Mater. Interfaces 2021, 13, 11344. 33620195 10.1021/acsami.1c01343

[6] X. He , B. Zhang , Q. Liu , H. Chen , J. Cheng , B. Jian , H. Yin , H. Li , K. Duan , J. Zhang , Q. Ge , Nat. Commun. 2024, 15, 6431. 39085229 10.1038/s41467-024-50797-w PMC11291765

[7] Z. Jiang , P. Song , Science 2022, 376, 245. 35420950 10.1126/science.abo4603

[8] W. Li , Q. Guan , M. Li , E. Saiz , X. Hou , Prog. Polym. Sci. 2023, 140, 101665.

[9] J. Ko , D. Kim , Y. Song , S. Lee , M. Kwon , S. Han , D. Kang , Y. Kim , J. Huh , J.‐S. Koh , J. Cho , ACS Nano 2020, 14, 11906. 32885947 10.1021/acsnano.0c04899

[10] Y. Liang , J. He , B. Guo , ACS Nano 2021, 15, 12687. 34374515 10.1021/acsnano.1c04206

[11] M. Shao , Z. Shi , X. Zhang , B. Zhai , J. Sun , Materials 2023, 16, 1358. 36836988 10.3390/ma16041358 PMC9967607

[12] C. Yu , Z. Yue , M. Shi , L. Jiang , S. Chen , M. Yao , Q. Yu , X. Wu , H. Zhang , F. Yao , C. Wang , H. Sun , J. Li , ACS Nano 2022, 16, 16234. 36190461 10.1021/acsnano.2c05168

[13] J. Wu , H. Yuk , T. L. Sarrafian , C. F. Guo , L. G. Griffiths , C. S. Nabzdyk , X. Zhao , Sci. Transl. Med. 2022, 14, eabh2857. 35108064 10.1126/scitranslmed.abh2857

[14] F. Wang , Y. Xue , X. Chen , P. Zhang , L. Shan , Q. Duan , J. Xing , Y. Lan , B. Lu , J. Liu , Adv. Funct. Mater. 2024, 34, 2314471.

[15] M. Xu , Q. Li , Z. Fang , M. Jin , Q. Zeng , G. Huang , Y.‐G. Jia , L. Wang , Y. Chen , Biomater. Sci. 2020, 8, 6957. 33103177 10.1039/d0bm01466d

[16] T. Qin , W. Liao , L. Yu , J. Zhu , M. Wu , Q. Peng , L. Han , H. Zeng , J. Polym. Sci. 2022, 60, 2607.

[17] Q. Liang , X. Xia , X. Sun , D. Yu , X. Huang , G. Han , S. M. Mugo , W. Chen , Q. Zhang , Adv. Sci. 2022, 9, 2201059. 10.1002/advs.202201059 PMC9165511 35362243

[18] M. Wen , T. Wang , N. Li , Y. Wu , L. Zhang , Y. Xue , L. Shang , Adv. Funct. Mater. 2024, 34, 2403634.

[19] Z. Li , J. Lu , T. Ji , Y. Xue , L. Zhao , K. Zhao , B. Jia , B. Wang , J. Wang , S. Zhang , Z. Jiang , Adv. Mater. 2024, 36, 2306350. 10.1002/adma.202306350 37987498

[20] X. Hu , F. Yang , M. Wu , Y. Sui , D. Guo , M. Li , Z. Kang , J. Sun , J. Liu , Adv. Mater. Technol. 2022, 7, 2100769.

[21] X. Peng , W. Wang , W. Yang , J. Chen , Q. Peng , T. Wang , D. Yang , J. Wang , H. Zhang , H. Zeng , J. Colloid Interface Sci. 2022, 618, 111. 35338921 10.1016/j.jcis.2022.03.037

[22] P. Jiang , H. Qin , J. Dai , S. Yu , H. Cong , Nano Lett. 2022, 22, 1433. 34747171 10.1021/acs.nanolett.1c03618

[23] J. He , Y. Sun , Q. Gao , C. He , K.e Yao , T. Wang , M. Xie , K. Yu , J. Nie , Y. Chen , Y. He , Adv. Healthcare Mater. 2023, 12, 2300395. 10.1002/adhm.202300395 37115708

[24] C. M. Lai , W. J. Chen , Y. Qin , D. Xu , Y. K. Lai , S. H. He , Adv. Sci. 2025, 12, 2412360. 10.1002/advs.202412360 PMC11727140 39575827

[25] B. Kong , Y. Chen , R. Liu , X. Liu , C. Liu , Z. Shao , L. Xiong , X. Liu , W. Sun , S. Mi , Nat. Commun. 2020, 11, 1435. 32188843 10.1038/s41467-020-14887-9 PMC7080797

[26] Z. Qin , H. Chen , Y. Fang , G. Wu , Q. Chen , B. Xue , R. Xu , K. Zheng , H. Jiang , ACS Appl. Mater. Interfaces 2024, 16, 55130. 39352138 10.1021/acsami.4c11767

[27] H. Cheng , Z. Shi , K. Yue , X. Huang , Y. Xu , C. Gao , Z. Yao , Y. S. Zhang , J. Wang , Acta Biomater. 2021, 124, 219. 33556605 10.1016/j.actbio.2021.02.002

[28] T. Wu , C. Cui , C. Fan , Z. Xu , Y. Liu , W. Liu , Bioact. Mater. 2021, 6, 2820. 33718664 10.1016/j.bioactmat.2021.02.009 PMC7903155

[29] B. Liu , Y. Wang , Y. Miao , X. Zhang , Z. Fan , G. Singh , X. Zhang , K. Xu , B. Li , Z. Hu , M. Xing , Biomaterials 2018, 171, 83. 29684678 10.1016/j.biomaterials.2018.04.023

[30] H. H. Hsu , Y. Liu , Y. Wang , B. Li , G. Luo , M. Xing , W. Zhong , ACS Sustainable Chem. Eng. 2020, 8, 6935.

[31] Z. Wang , G. An , Y. Zhu , X. Liu , Y. Chen , H. Wu , Y. Wang , X. Shi , C. Mao , Mater. Horiz. 2019, 6, 733. 31572613 10.1039/C8MH01208C PMC6768557

[32] S. Wang , J. Lei , X. Yi , L. Yuan , L. Ge , D. Li , C. Mu , ACS Appl. Polym. Mater. 2020, 2, 3016.

[33] J. Chen , J. He , Y. Yang , L. Qiao , J. Hu , J. Zhang , B. Guo , Acta Biomater. 2022, 146, 119. 35483628 10.1016/j.actbio.2022.04.041

[34] Q. Liu , J. Yang , Y. Wang , T. Wu , Y. Liang , K. Deng , G. Luan , Y. Chen , Z. Huang , K. Yue , Biomacromolecules 2023, 24, 2549. 37115848 10.1021/acs.biomac.3c00057

[35] W. Xu , W. Wang , S. Chen , R. Zhang , Y. Wang , Q. Zhang , L. Yuwen , W. J. Yang , L. Wang , J. Colloid Interface Sci. 2021, 586, 601. 33189325 10.1016/j.jcis.2020.10.128

[36] T. J. Long , Y. X. Li , X. Fang , J. Q. Sun , Adv. Funct. Mater. 2018, 28, 1804416.

[37] Y. Piao , H. You , T. Xu , H.‐P. Bei , I. Z. Piwko , Y. Y. Kwan , X. Zhao , Eng. Regener. 2021, 2, 47.

[38] D. T. Nguyen , H. N. Tran , R.‐S. Juang , N. D. Dat , F. Tomul , A. Ivanets , S. H. Woo , A. Hosseini‐Bandegharaei , V. P. Nguyen , H.‐P. Chao , J. Environ. Chem. Eng. 2020, 8, 104408.

[39] L. Chen , J. Shao , Q. Yu , S. Wang , J. Dispersion Sci. Technol. 2020, 43, 690.

[40] E. Mitani , Y. Ozaki , H. Sato , Polymer 2022, 246, 124725.

[41] B. Lu , F. Lin , X. Jiang , J. Cheng , Q. Lu , J. Song , C. Chen , B. Huang , ACS Sustainable Chem. Eng. 2017, 5, 948.

[42] M. Tavafoghi , A. Sheikhi , R. Tutar , J. Jahangiry , A. Baidya , R. Haghniaz , A. Khademhosseini , Adv. Healthcare Mater. 2020, 9, 1901722. 10.1002/adhm.201901722 PMC9386893 32329254

[43] H. Montazerian , A. Baidya , R. Haghniaz , E. Davoodi , S. Ahadian , N. Annabi , A. Khademhosseini , P. S. Weiss , ACS Appl. Mater. Interfaces 2021, 13, 40290. 34410697 10.1021/acsami.1c10048

[44] H. Tang , Y. Li , B. Chen , X. Chen , Y. Han , M. Guo , H.‐Q. Xia , R. Song , X. Zhang , J. Zhou , ACS Nano 2022, 16, 17931. 36200714 10.1021/acsnano.2c03414

[45] Y. Huang , H. Zhao , X. Wang , X. Liu , Z. Gao , H. Bai , F. Lv , Q. Gu , S. Wang , Chem. Commun. 2022, 58, 6894. 10.1039/d2cc02176e 35638877

[46] M. Hafezi , S. N. Khorasani , S. Khalili , R. E. Neisiany , Int. J. Biol. Macromol. 2023, 242, 124962. 37207752 10.1016/j.ijbiomac.2023.124962

[47] C. Liu , Q. Yu , Z. Yuan , Q. Guo , X. Liao , F. Han , T. Feng , G. Liu , R. Zhao , Z. Zhu , H. Mao , C. Zhu , B. Li , Bioact. Mater. 2023, 25, 445. 37056254 10.1016/j.bioactmat.2022.07.031 PMC10087107

[48] Z. Qin , X. Sun , H. Zhang , Q. Yu , X. Wang , S. He , F. Yao , J. Li , J. Mater. Chem. A 2020, 8, 4447.

[49] J. Yin , C. Lu , C. Li , Z. Yu , C. Shen , Y. Yang , X. Jiang , Y. Zhang , Compos. Part B‐Eng. 2022, 230, 109528.

[50] S. Q. Guan , C. Xu , X. F. Dong , M. Qi , J. Mater. Chem. A 2023, 11, 15404.

[51] W. Zhao , X. Qu , Q. Xu , Y. Lu , W. Yuan , W. Wang , Q. Wang , W. Huang , X. Dong , Adv. Electron. Mater. 2020, 6, 2000267.

[52] Z. Bian , Y. Li , H. Sun , M. Shi , Y. Zheng , H. Liu , C. Liu , C. Shen , Carbohydr. Polym. 2023, 301, 120300. 36436853 10.1016/j.carbpol.2022.120300

[53] L. Guan , H. Liu , X. Ren , T. Wang , W. Zhu , Y. Zhao , Y. Feng , C. Shen , A. V. Zvyagin , L. Fang , B. Yang , Q. Lin , Adv. Funct. Mater. 2022, 32, 2112281.

[54] H. X. Luan , D. Z. Zhang , Z. Y. Xu , W. H. Zhao , C. Q. Yang , X. Y. Chen , J. Mater. Chem. C 2022, 10, 7604.

[55] J. Yang , Z. Liu , K. Li , J. Hao , Y. Guo , M. Guo , Z. Li , S. Liu , H. Yin , X. Shi , G. Qin , G. Sun , L. Zhu , Q. Chen , ACS Appl. Mater. Interfaces 2022, 14, 39299. 35972900 10.1021/acsami.2c07213

[56] Y. Niu , H. Liu , R. Y. He , M. Q. Luo , M. G. Shu , F. Xu , Small 2021, 17, 2101151. 10.1002/smll.202101151 34013638

