# Supplementary material for: Highly Stretchable, Self‐Healable, and Conductive Gelatin Methacryloyl Hydrogel for Long‐Lasting Wearable Tactile Sensors
Source: Adv Sci (Weinh). 2025 May 29;12(30):e02678. doi: 10.1002/advs.202502678 (PMC12376516; doi:10.1002/advs.202502678)
Supplement: Supplementary file 1 — Supporting Information [file ADVS-12-e02678-s002.docx]

Supporting Information

Highly Stretchable, Self-healable, and Conductive Gelatin Methacryloyl Hydrogel for Long-Lasting Wearable Tactile Sensors

Zhikang Li, Bin Wang, Jijian Lu, Yumeng Xue*, Jiaxiang Wang, Boqing Jia, Gengyu Han*, Yihe Zhao, Muhammad Afzal Khan Qureshi, Lan Yu, Kang Zhao, Min Li, Ping Yang, Dejiang Lu, Libo Zhao*

**Experimental Section**

***Synthesis of GelMA***: GelMA was synthesized according to the previously established method with minor modifications.^[1][2]^ Initially, 10 g of gelatin was dissolved in 100 mL of DPBS at 50 ℃ to obtain a homogeneous gelatin solution. Then, 8 mL of MA was added to the gelatin solution drop by drop under stirring and allowed to react for 2 h at 50 ℃. Afterward, another 100 mL of DPBS that was pre-heated to 50 ℃ was added to the mixed solution to halt the reaction. The resultant solution was subdivided into 12-14 kDa dialysis tubes and dialyzed in DI water for at least a week to remove the unreacted MA and other by-products. Furthermore, the GelMA solution after dialysis was lyophilized at -80 ℃ for at least 5 days to obtain porous foam solid material and stored at -20 ℃ for further use. The degree of MA substitution (DS) was calculated to be 85% based on the 1H NMR spectrum of GelMA and gelatin.

***Preparation of gelatin/PAA/PSBMA (GPP) hydrogel glue***: The gelatin/AA/SBMA precursor solution was prepared by a simple one-pot method, composed of 10% w/v gelatin and SBMA, 20% v/v AA, and 1% w/v Irgacure 2959 photoinitiator. Then, the precursor solution could be polymerized further into gelatin/PAA/PSBMA (GPP) hydrogel glue under UV light irradiation.

***Mechanical Property Measurement***: The tensile testing speed was set at 50 mm·min^-1^ at room temperature (25 ℃). The tensile elastic modulus (E) was defined as the fit slope of the tensile stress-strain curves (0-20% strain). The toughness (Γ) was calculated by integrating the area of tensile stress-strain curves, and the equation is

$\Gamma=\int_{\varepsilon_{0}}^{\varepsilon_{f}} \sigma d\varepsilon$ (1)

Where $\varepsilon_{0}$ and $\varepsilon_{f}$ refer to the initial strain and fractured strain, respectively. The energy dissipation (Δ*U_i_*) was calculated by integrating the area of the loading-unloading stress-strain curve at the *i* time, and the equation is

$\Delta U_{i}=\int_{loading} \sigma d\varepsilon-\int_{unloading} \sigma d\varepsilon$ (2)

The energy dissipated ratio (δ) was calculated by the equation

$\delta=\Delta U_{i}/U_{i}$ (3)

$U_{i}=\int_{loading} \sigma d\varepsilon$ (4)

The adhesive strength between GNPB hydrogel and Cu tape was measured by lap-shear testing. All the tests were conducted at least 3 times for each group.

**
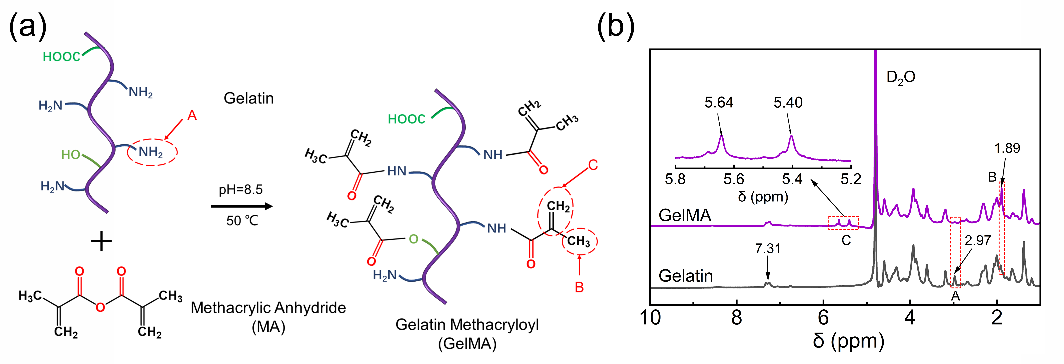
**

**Figure S1.** (a) Schematic of the synthetic process of GelMA, (b) ^1^H NMR spectrum of the gelatin and GelMA.


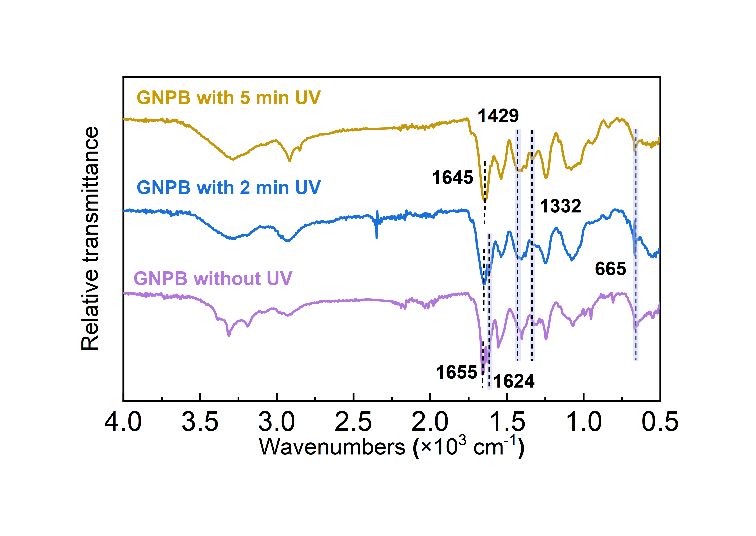


**Figure S2.** FTIR spectrum of GNPB hydrogels without UV illumination, with UV illumination for 2 minutes, and with UV illumination for 5 minutes.


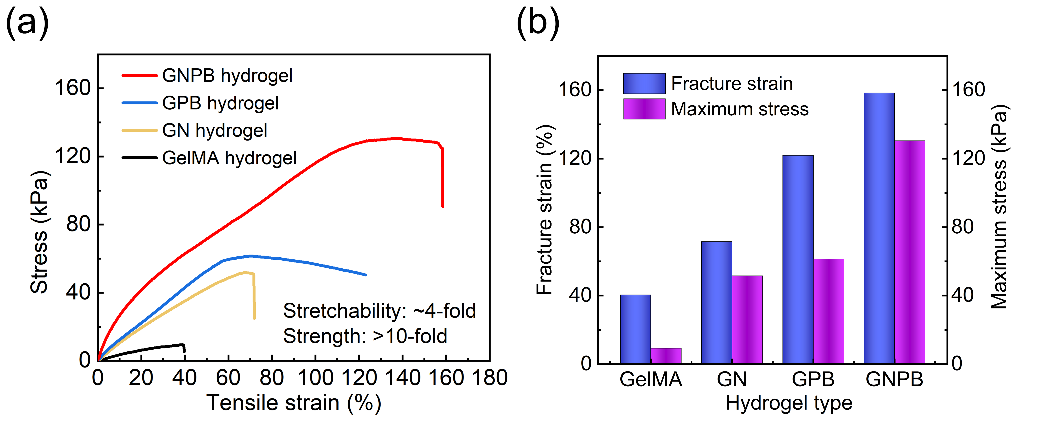


**Figure S3.** The comparison of the tensile properties of pure GelMA hydrogel, GN hydrogel, GPB hydrogel, and GNPB hydrogel. (a) Tensile stress-strain curve. (b) The corresponding fracture strain and maximum stress.


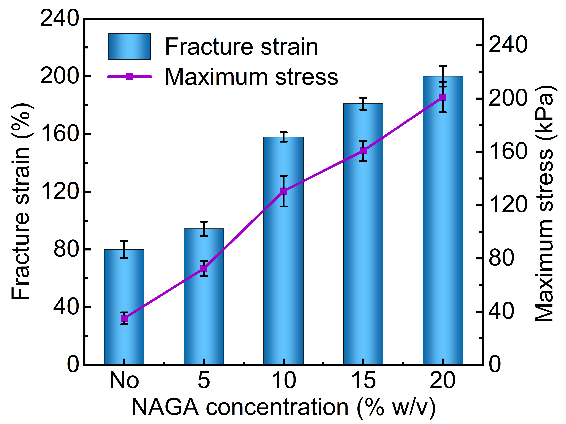


**Figure S4.** The corresponding fracture strain and maximum stress of GNPB hydrogels with different NAGA concentrations.


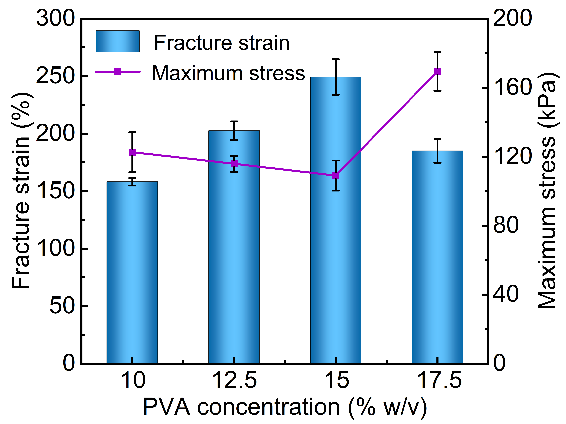


**Figure S5.** The corresponding fracture strain and maximum stress of the GNPB hydrogel with different PVA concentrations.


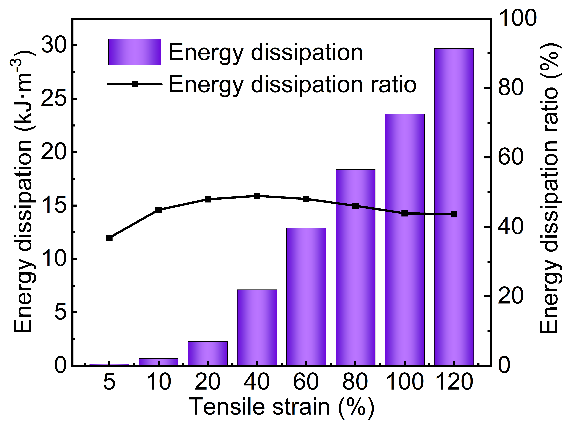


**Figure S6.** The corresponding energy dissipation and energy dissipation ratio of the optimized GNPB hydrogel from 5% strain to 120% strain.


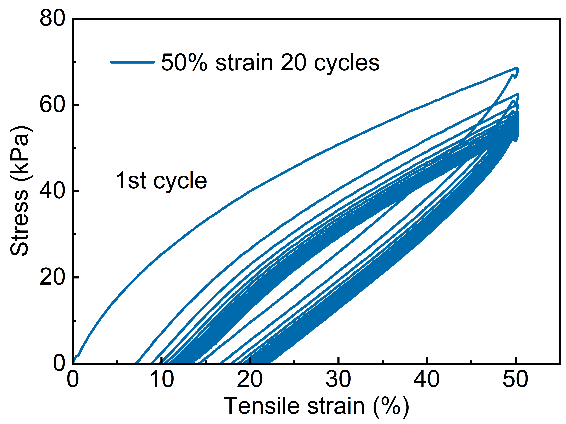


**Figure S7.** The strain-stress curves of 20 loading-unloading cycles at 50% strain of the optimized GNPB hydrogel.


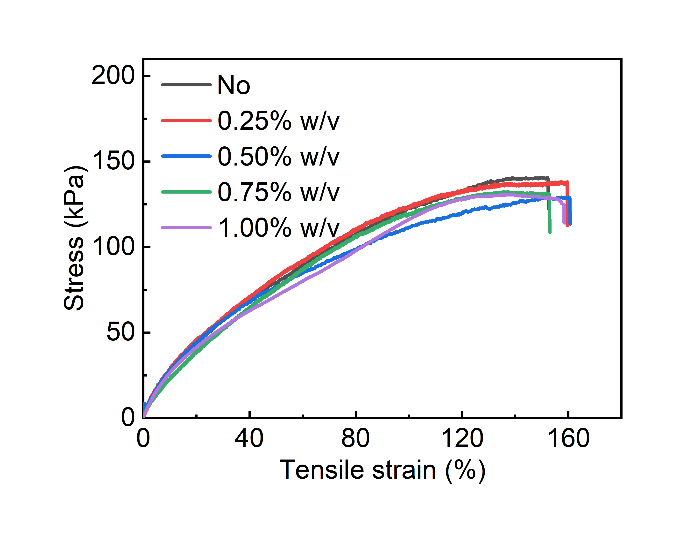


**Figure S8.** The tensile properties of GNPB hydrogels with different NaCl concentrations.


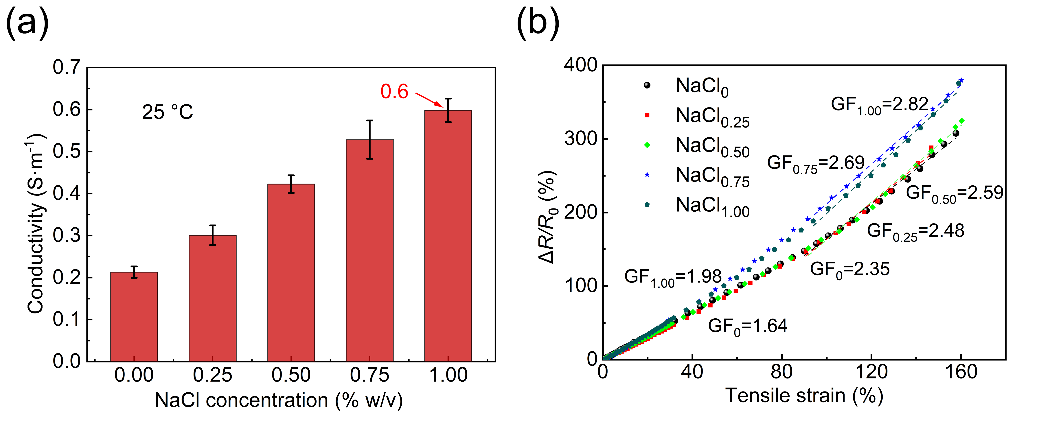


**Figure S9.** The conductivity and sensitivity of the GNPB hydrogels with different NaCl concentrations.


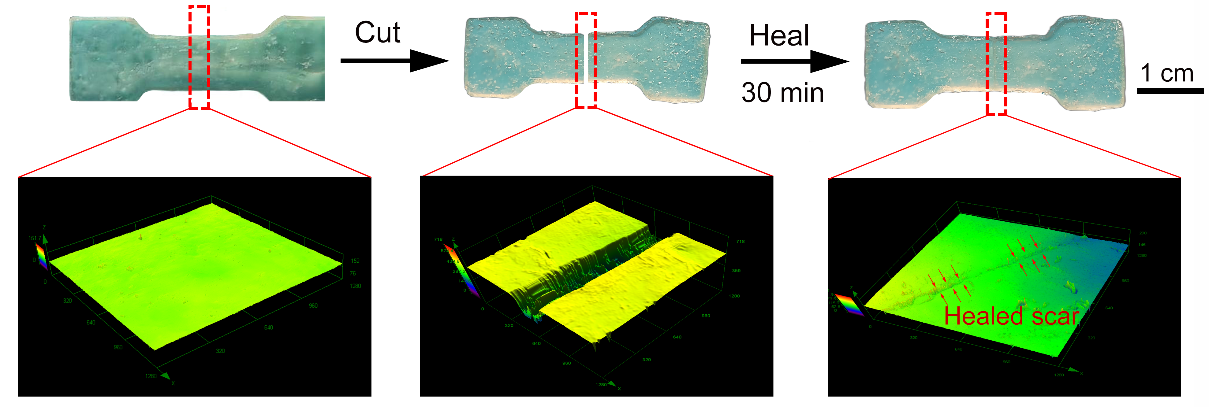


**Figure S10.** The laser scanning confocal microscope (LSCM) images of the original hydrogel’s surface and the cracked and healed interfaces.


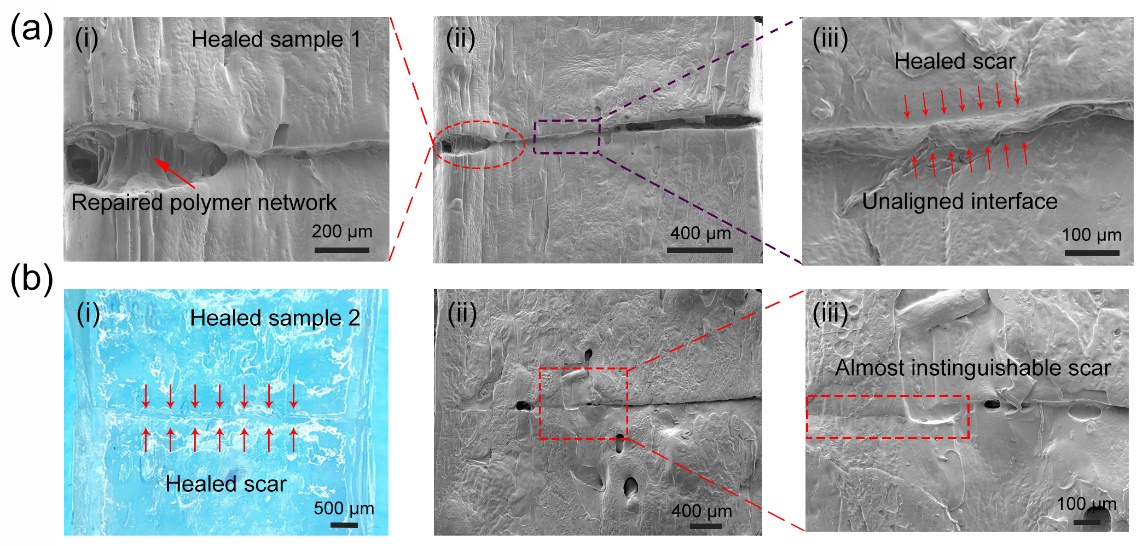


**Figure S11.** The scanning electron microscope (SEM) images of the healed interfaces of the different healed samples. (a) The healed sample with the unaligned interfaces. (b) The healed sample with better healing interfaces.


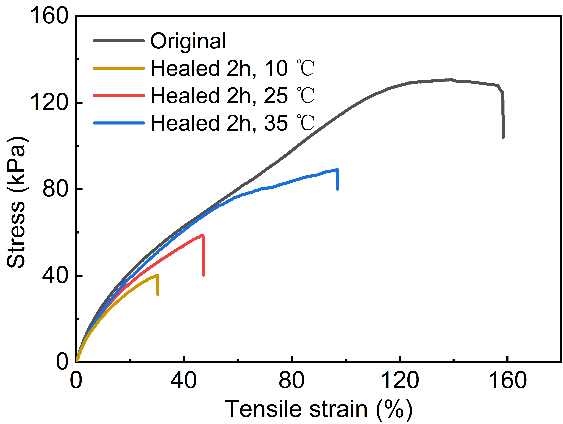


**Figure S12.** Tensile stress-strain curves of GNPB hydrogels under different healing temperatures.


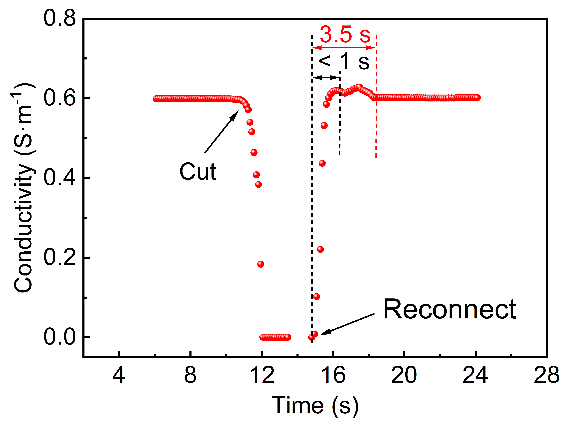


**Figure S13.** The self-healing speed and stable time of the conductivity.


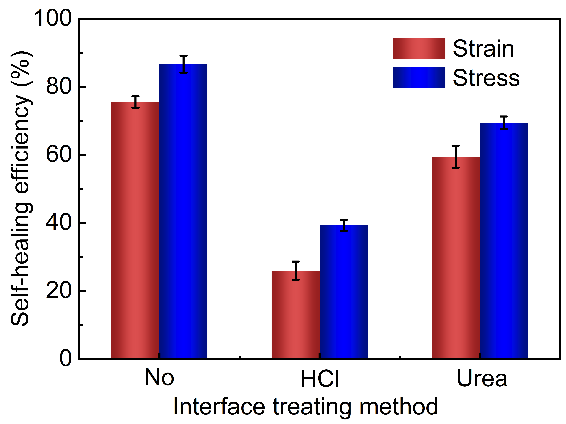


**Figure S14.** The corresponding self-healing efficiencies of the healed hydrogels with 48 h of healing time after treating the fractured surface in different ways.


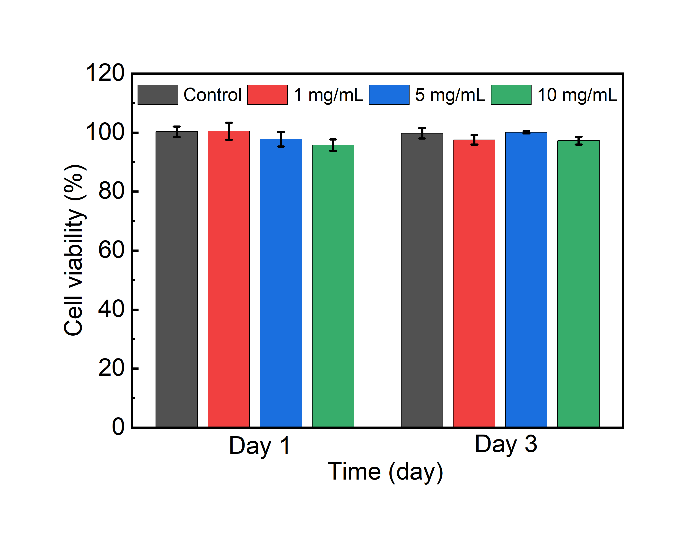


**Figure S15.** The cell viability under the culture of different concentrations of GNPB hydrogel extracts (n = 3).


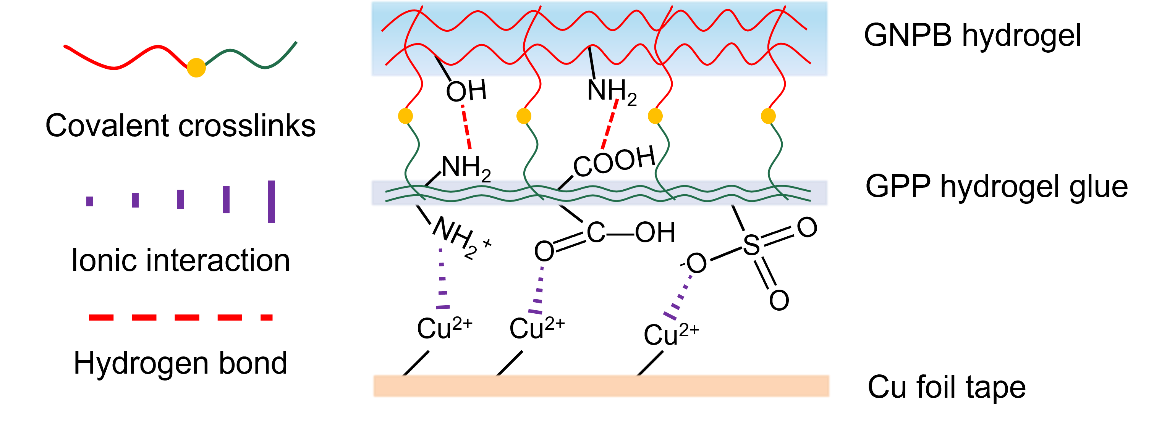


**Figure S16.** Mechanism schematic of the adhesive interactions between GNPB hydrogel and GPP hydrogel glue, GPP hydrogel glue, and Cu foil tape.


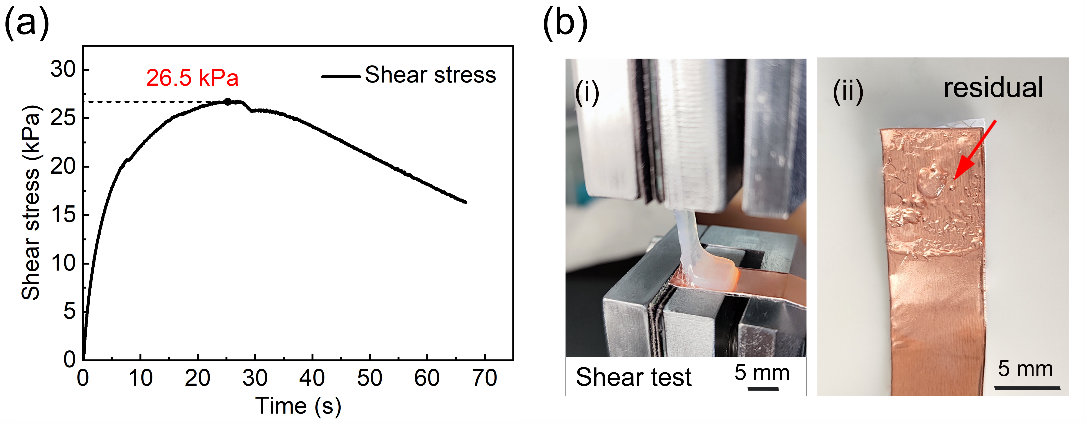


**Figure S17.** The test of the adhesive strength between GNPB hydrogel and Cu tape. (a) The shear stress-strain curve. (b) Demonstration of the shear test (i) and the photos of hydrogel residue (ii).


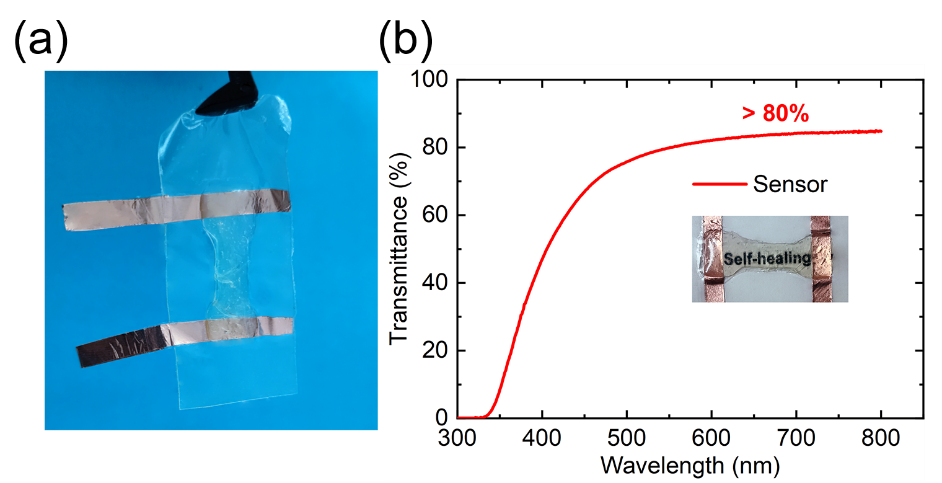


**Figure S18.** (a) The as-prepared sensor’s photos. (b) The transmittance of the GNPB hydrogel-based sensor.


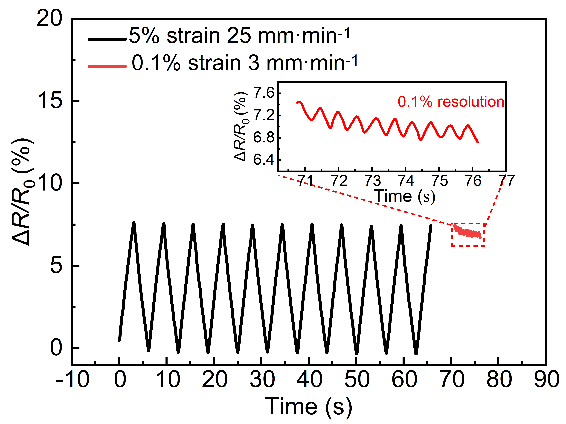


**Figure S19.** The relative resistance change (ΔR/R_0_) curves at the strain of 5% and from 5% strain to 5.1% strain.


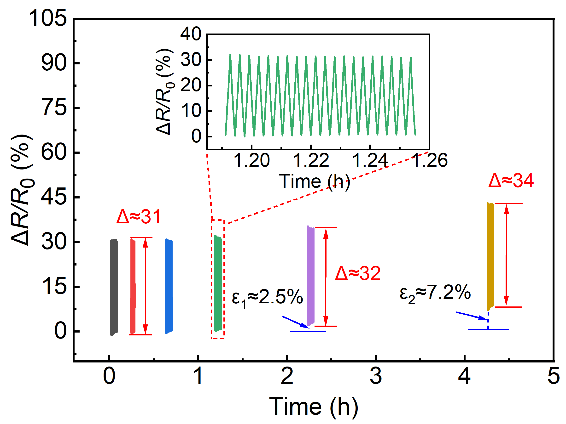


**Figure S20.** The stability testing of the sensor at 20% tensile strain in 4 hours.


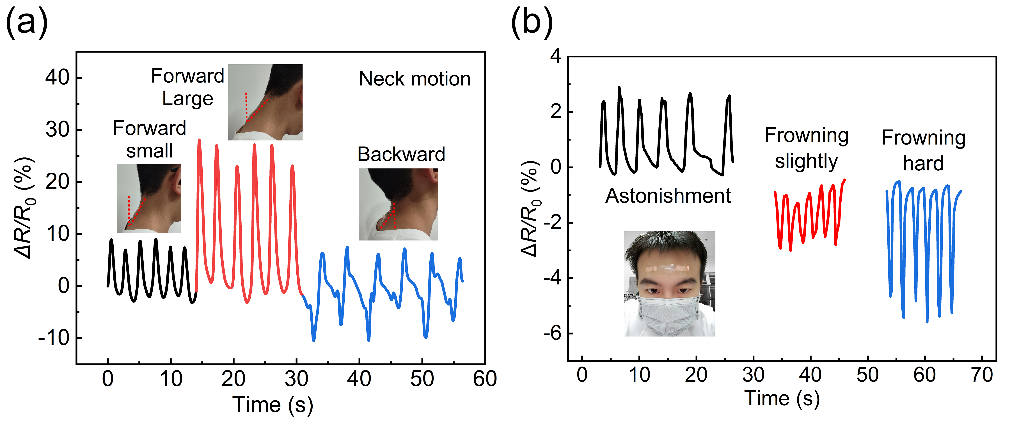


**Figure S21.** The relative resistance change (ΔR/R_0_) curves of (a) neck motion and (b) facial expression detection.


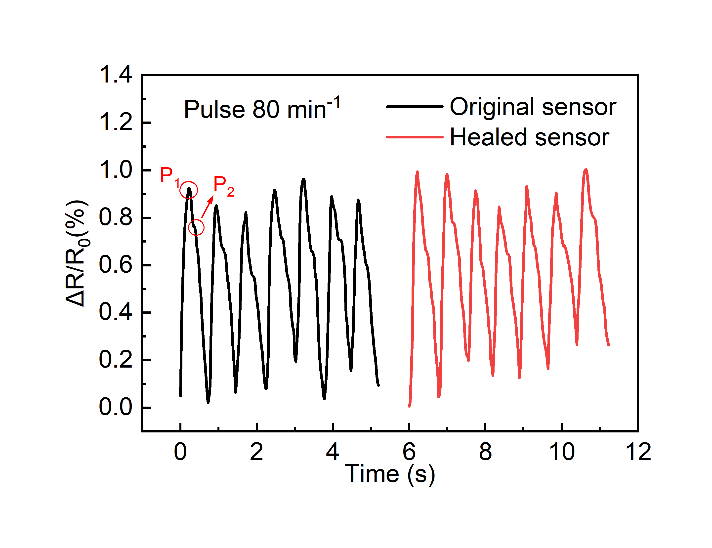


**Figure S22.** The recognition of pulse signals by original and healed sensors.


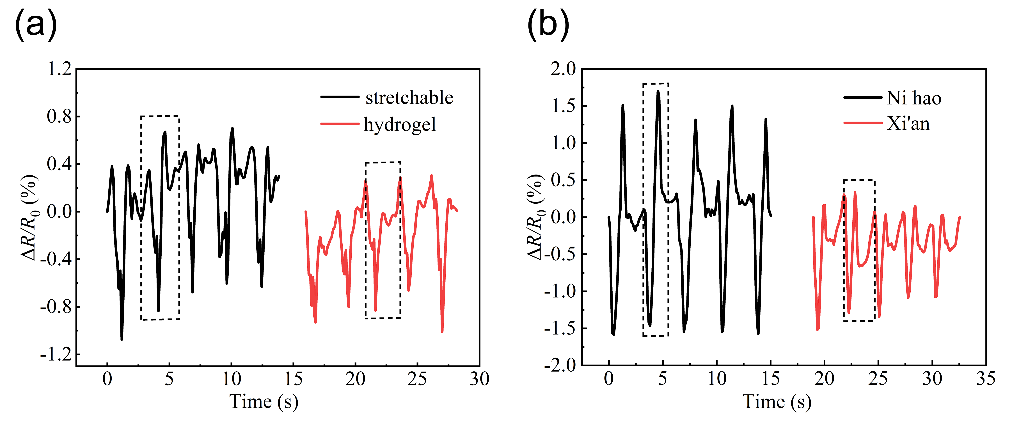


**Figure S23.** The recognition of the word (a) ‘Stretchable’ and ‘Hydrogel’, (b) ‘Ni hao’ and ‘Xi’an’.


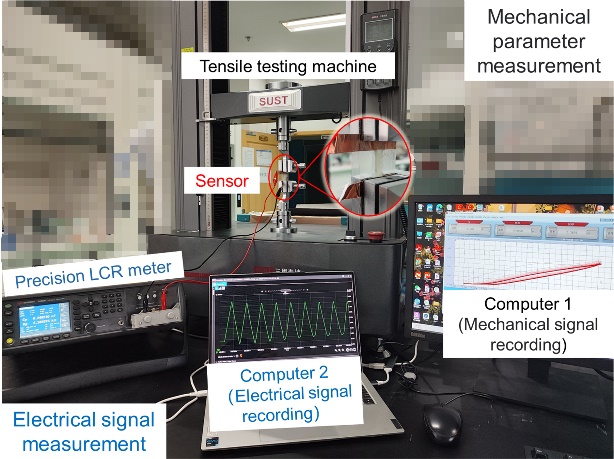


**Figure S24.** The testing system of the GNPB hydrogel-based strain sensor.

**Table S1.** Comparison of the GNPB hydrogel with other GelMA-based hydrogels

| **Materials** | **Stretchability**  **(%)** | **Tensile strength**  **(kPa)** | **Self-healing** | **Ref.** |
| --- | --- | --- | --- | --- |
| GelMA/NAGA/  PVA/Borax/NaCl | ~160 | 130 | 76%, 48 h  strain | This work |
| GelMA+AlgMA | ~75 | ~105 | No | [[3](#_ENREF_3)] |
| Dopamine@GelMA | ~140 | ~30 | No | [[4](#_ENREF_4)] |
| GelMA/TA | 227 | 150 | Macro mechanical | [[5](#_ENREF_5)] |
| GelMA/Chitosan | 96 | 19.25 | No | [[6](#_ENREF_6)] |
| GelMA/PEGDA/  PDA/CNTs | 100 | 20 | No | [[7](#_ENREF_7)] |
| GelMA+AA+Cu | 124.6 | 92 | Macro mechanical | [[8](#_ENREF_8)] |
| GelMA/A-TEG-Ad/  β-CD-AOI_2_ | 70 | 225 | 80%, 1 h  strain | [[9](#_ENREF_9)] |
| GelMA/SilMA/F127 DA micelles | 126.3 | 78.7 | No | [[10](#_ENREF_10)] |
| GelMA/PU/HEMA | 76 | 120 | No | [[1](#_ENREF_11)1] |
| GelMA/Algin/NC | ~120 | ~31.5 | ~52%, strain | [12] |
| GelMA/PEDOT: PSS | ~80 | 45.2 | No | [[1](#_ENREF_13)3] |
| GelMA/gelatin-PBA/DB | 65 | 15 | No | [[1](#_ENREF_14)4] |

**Table S2.** In comparison with other natural polymers hydrogel-based strain sensor

| **Materials** | **Self-healing efficiency** | **Conductivity (S·m^-1^)** | **GF** | **LOD**  **(%)** | **Response/recovery time (ms)** | **Durability**  **(cycles)** | **Ref.** |
| --- | --- | --- | --- | --- | --- | --- | --- |
| GelMA/NAGA/PVA/Borax/NaCl | 76% (strain)  48 h | 0.6 | 1.98 (0~80%) | 0.1 | 257/264 | 800 | This work |
| Gelatin/Na_3_Cit/  glycerol | No | 0.47 | 1.5 | 5.0 | 200/400 | 100 | [[1](#_ENREF_15)5] |
| PVA/gelatin/  TA@CNC–Al^3+^ | 69.3% (strain) | 0.23 | 1.86 (0~100%) | 1 | 240/280 | 1000 | [16] |
| HA/PAAm/Zn^2+^ | No | 0.44 | 0.52 (0~100%) | 1 | 382/488 | 500 | [17] |
| CMC/PVA/AA/  AgNWs | 94.3% (strain)  72 h | 0.69 | 0.53 (0~150%) | 4 | -/300 | 80 | [18] |
| CCNF/PEDOT: PSS/PAAm | No | 0.058 | 0.16 (0~160%) | 1 | 400/400 | 1100 | [19] |
| GA/HK/Mxene/  PAAm/LMA | ~11% (strain) | 0.364 | 1.79 (0~100%) | 1 | 130/200 | 200 | [20] |
| SA/PAAm/MXene | No | 0.16 | 0.81 (0~100%) | 5 | 750/810 | 300 | [21] |
| BSA/PAAm/ glycerol | No | 0.025 | 0.973 (0~100%) | 10 | 500/- | 300 | [22] |
| Gelatin/PAA/NHS ester/LiCl/glycerol | No | 0.6 | 0.23 (0~300%) | 10 | 116/68 | 1200 | [23] |

Note: “-” means “not shown” in the reference.

**References**

[1] Nichol, J. W., Koshy, S. T., Bae, H., Hwang, C. M., Yamanlar, S., Khademhosseini, A., Cell-Laden Microengineered Gelatin Methacrylate Hydrogels, 2010, Biomaterials, 31, 5536, https://doi.org/10.1016/j.biomaterials.2010.03.064

[2] Van Den Bulcke, A. I., Bogdanov, B., De Rooze, N., Schacht, E. H., Cornelissen, M., Berghmans, H., Structural and Rheological Properties of Methacrylamide Modified Gelatin Hydrogels, 2000, Biomacromolecules, 1, 31, https://doi.org/10.1021/bm990017d

[3] Tavafoghi, M., Sheikhi, A., Tutar, R., et al., Engineering Tough, Injectable, Naturally Derived, Bioadhesive Composite Hydrogels, 2020, Adv. Healthcare Mater., 9, 1901722, https://doi.org/10.1002/adhm.201901722

[4] Montazerian, H., Baidya, A., Haghniaz, R., et al., Stretchable and Bioadhesive Gelatin Methacryloyl-Based Hydrogels Enabled by in Situ Dopamine Polymerization, 2021, ACS Appl. Mater. Interfaces, 13, 40290, https://doi.org/10.1021/acsami.1c10048

[5] Liu, B., Wang, Y., Miao, Y., et al., Hydrogen Bonds Autonomously Powered Gelatin Methacrylate Hydrogels with Super-Elasticity, Self-Heal and Underwater Self-Adhesion for Sutureless Skin and Stomach Surgery and E-skin, 2018, Biomaterials, 171, 83, https://doi.org/10.1016/j.biomaterials.2018.04.023

[6] Suo, H., Zhang, D., Yin, J., Qian, J., Wu, Z. L., Fu, J., Interpenetrating Polymer Network Hydrogels Composed of Chitosan and Photocrosslinkable Gelatin with Enhanced Mechanical Properties for Tissue Engineering, 2018, Mater. Sci. Eng. C, 92, 612, https://doi.org/10.1016/j.msec.2018.07.016

[7] Tang, H., Li, Y., Chen, B., et al., In Situ Forming Epidermal Bioelectronics for Daily Monitoring and Comprehensive Exercise, 2022, ACS Nano, 16, 17931, https://doi.org/10.1021/acsnano.2c03414

[8] Chen, J., He, J., Yang, Y., et al., Antibacterial Adhesive Self-Healing Hydrogels to Promote Diabetic Wound Healing, 2022, Acta Biomater., 146, 119, https://doi.org/10.1016/j.actbio.2022.04.041

[9] Wang, Z., An, G., Zhu, Y., et al., 3D-Printable Self-Healing and Mechanically Reinforced Hydrogels with Host–Guest Non-Covalent Interactions Integrated into Covalently Linked Networks, 2019, Mater. Horiz., 6, 733, https://doi.org/10.1039/c8mh01208c

[10] Fu, Z., Xiao, S., Wang, P., et al., Injectable, Stretchable, Toughened, Bioadhesive Composite Hydrogel for Bladder Injury Repair, 2023, RSC Adv., 13, 10903, https://doi.org/10.1039/d3ra00402c

[11] Huang, Y., Zhao, H., Wang, X., et al., Polyurethane–Gelatin Methacryloyl Hybrid Ink for 3D Printing of Biocompatible and Tough Vascular Networks, 2022, Chem. Commun., 58, 6894, https://doi.org/10.1039/d2cc02176e

[12] Hafezi, M., Khorasani, S. N., Khalili, S., Neisiany, R. E., Self-Healing Interpenetrating Network Hydrogel Based on GelMA/Alginate/Nnano-Clay, 2023, Int. J. Biol. Macromol., 242, 124962, https://doi.org/10.1016/j.ijbiomac.2023.124962

[13] Lee, J., Jr., Ng, H. Y., Lin, Y.-H., et al., The 3D Printed Conductive Grooved Topography Hydrogel Combined with Electrical Stimulation for Synergistically Enhancing Wound Healing of Dermal Fibroblast Cells, 2022, Biomater. Adv., 142, 213132, https://doi.org/10.1016/j.bioadv.2022.213132

[14] Liu, C., Yu, Q., Yuan, Z., et al., Engineering the Viscoelasticity of Gelatin Methacryloyl (GelMA) Hydrogels Via Small “Dynamic Bridges” to Regulate BMSC Behaviors for Osteochondral Regeneration, 2023, Bioact. Mater., 25, 445, https://doi.org/10.1016/j.bioactmat.2022.07.031

[15] Qin, Z., Sun, X., Zhang, H., et al., A Transparent, Ultrastretchable and Fully Recyclable Gelatin Organohydrogel Based Electronic Sensor with Broad Operating Temperature, 2020, J. Mater. Chem. A, 8, 4447, https://doi.org/10.1039/c9ta13196e

[16] Yin, J., Lu, C., Li, C., et al., A UV-Filtering, Environmentally Stable, Healable and Recyclable Ionic Hydrogel Towards Multifunctional Flexible Strain Sensor, 2022, Compos. Part B-Eng., 230, 109528, https://doi.org/10.1016/j.compositesb.2021.109528

[17] Guan, S., Xu, C., Dong, X., Qi, M., Highly Tough, Fatigue-Resistant, Low Hysteresis Hybrid Hydrogel with Hierarchical Cross-Linked Structure for Wearable Strain Sensor, 2023, J. Mater. Chem. A, 11, 15404, https://doi.org/10.1039/d3ta02584e

[18] Zhao, W., Qu, X., Xu, Q., et al., Ultrastretchable, Self‐Healable, and Wearable Epidermal Sensors Based on Ultralong Ag Nanowires Composited Binary‐Networked Hydrogels, 2020, Adv. Electron. Mater., 6, 2000267, https://doi.org/10.1002/aelm.202000267

[19] Bian, Z., Li, Y., Sun, H., et al., Transparent, Intrinsically Stretchable Cellulose Nanofiber-Mediated Conductive Hydrogel for Strain and Humidity Sensing, 2023, Carbohydr. Polym., 301, 120300, https://doi.org/10.1016/j.carbpol.2022.120300

[20] Guan, L., Liu, H., Ren, X., et al., Balloon Inspired Conductive Hydrogel Strain Sensor for Reducing Radiation Damage in Peritumoral Organs During Brachytherapy, 2022, Adv. Funct. Mater., 32, 2112281, https://doi.org/10.1002/adfm.202112281

[21] Luan, H., Zhang, D., Xu, Z., Zhao, W., Yang, C., Chen, X., MXene-Based Composite Double-Network Multifunctional Hydrogels as Highly Sensitive Strain Sensors, 2022, J. Mater. Chem. C, 10, 7604, https://doi.org/10.1039/d2tc00679k

[22] Yang, J., Liu, Z., Li, K., et al., Tough Adhesive, Antifreezing, and Antidrying Natural Globulin-Based Organohydrogels for Strain Sensors, 2022, ACS Appl. Mater. Interfaces, 14, 39299, https://doi.org/10.1021/acsami.2c07213

[23] Niu, Y., Liu, H., He, R., Luo, M., Shu, M., Xu, F., Environmentally Compatible Wearable Electronics Based on Ionically Conductive Organohydrogels for Health Monitoring with Thermal Compatibility, Anti‐Dehydration, and Underwater Adhesion, 2021, Small, 17, 2101151, https://doi.org/10.1002/smll.202101151
